# Palmitoylation Regulates Epidermal Homeostasis and Hair Follicle Differentiation

**DOI:** 10.1371/journal.pgen.1000748

**Published:** 2009-11-26

**Authors:** Pleasantine Mill, Angela W. S. Lee, Yuko Fukata, Ryouhei Tsutsumi, Masaki Fukata, Margaret Keighren, Rebecca M. Porter, Lisa McKie, Ian Smyth, Ian J. Jackson

**Affiliations:** 1 Medical Research Council, Human Genetics Unit, Edinburgh, United Kingdom; 2 National Institute for Physiological Sciences, National Institutes of Natural Sciences, Okazaki, Japan; 3 Precursory Research for Embryonic Science and Technology, Japan Science and Technology Agency, Chiyoda, Tokyo, Japan; 4 Department of Dermatology, School of Medicine, Cardiff University, Cardiff, United Kingdom; 5 Cutaneous Developmental Biology Lab, Department of Biochemistry and Molecular Biology, Department of Anatomy and Developmental Biology, Monash University, Melbourne, Australia; Harvard Medical School, United States of America

## Abstract

Palmitoylation is a key post-translational modification mediated by a family of DHHC-containing palmitoyl acyl-transferases (PATs). Unlike other lipid modifications, palmitoylation is reversible and thus often regulates dynamic protein interactions. We find that the mouse hair loss mutant, depilated, (*dep*) is due to a single amino acid deletion in the PAT, Zdhhc21, resulting in protein mislocalization and loss of palmitoylation activity. We examined expression of Zdhhc21 protein in skin and find it restricted to specific hair lineages. Loss of Zdhhc21 function results in delayed hair shaft differentiation, at the site of expression of the gene, but also leads to hyperplasia of the interfollicular epidermis (IFE) and sebaceous glands, distant from the expression site. The specific delay in follicle differentiation is associated with attenuated anagen propagation and is reflected by decreased levels of Lef1, nuclear β-catenin, and Foxn1 in hair shaft progenitors. In the thickened basal compartment of mutant IFE, phospho-ERK and cell proliferation are increased, suggesting increased signaling through EGFR or integrin-related receptors, with a parallel reduction in expression of the key differentiation factor Gata3. We show that the Src-family kinase, Fyn, involved in keratinocyte differentiation, is a direct palmitoylation target of Zdhhc21 and is mislocalized in mutant follicles. This study is the first to demonstrate a key role for palmitoylation in regulating developmental signals in mammalian tissue homeostasis.

## Author Summary

During embryonic development, growth and patterning are regulated at many levels. Signals that mediate transcriptional activity, where and when genes are expressed, are a primary level of regulation. However, developmental signals can be further fine-tuned by modulating protein stability, localization, and activity via post-translational modifications. One such modification is the reversible addition of the fatty acid palmitate to proteins. This modification mediates dynamic trafficking of target proteins to specific subdomains of the cell. A large family of enzymes carries out this palmitoylation process, where each family member has specificity towards particular targets. However, the functional significance of palmitoylation during mammalian development is unclear. We present evidence of a critical role for palmitoylation during mouse development using a mutation of a specific palmitoylating enzyme, whose loss of function leads to hair loss and skin defects in depilated (*dep*) mice. Despite its restricted expression in hair follicles, loss of function of this enzyme results in developmental defects in nearby structures. We show that palmitoylation plays an important regulatory role in hair growth and epidermal homeostasis.

## Introduction

Palmitoylation (or protein *S*-acylation) is a reversible post-translational lipid modification which involves addition of the fatty acid palmitate onto specific cysteine residues [1]. Some post-translational lipid modifications such as myristoylation and prenylation serve to localize otherwise soluble proteins to the cytoplasmic surfaces of cellular membranes. In contrast, palmitoylation substrates are proteins that are already membrane associated, and the modification acts to increase or stabilise membrane affinity or to traffic the protein to specific membrane domains. In particular, palmitoylation results in localization of the protein to lipid rafts; domains of the plasma membrane rich in cholesterol and sphingolipids. Furthermore, as palmitoylation is reversible, it allows for membrane localization or trafficking to be dynamically regulated. This has best been demonstrated in synapses, where palmitoylation regulates membrane localization and activity of the AMPA receptor [2] and GABA_A_ receptor [3]. Palmitoylation of the post-synaptic density protein PSD95 permits clustering of the protein at synapses and regulates synaptic strength [4]. A recent global study of the neural palmitoyl-proteome highlights the breadth of targets that are rapidly modulated by palmitoylation [5], further emphasizing the importance of this modification in dynamic biological processes.

Members of the zinc finger, DHHC containing (ZDHHC) protein family have recently been shown to promote palmitoylation of intracellular proteins in yeast and in mammalian cells [6]–[8]. These palmitoyl-acyl transferases (PATs) are predicted membrane proteins possessing a cysteine-rich domain and a putative zinc finger with a characteristic Asp-His-His-Cys (DHHC) motif, required for activity. This family is encoded by 24 genes in both mouse and humans, of which 23 are orthologous pairs. Assaying individual target proteins against the entire repertoire of PATs indicates that there is substrate specificity; each substrate is primarily modified by a subgroup of structurally similar ZDHHC proteins [9].

Although some human *ZDHHC* genes have been implicated in cancer [10],[11], genetic evidence for function of these genes is limited to neurological disorders. *ZDHHC8* shows association with schizophrenia in humans and neurophysiological deficits in mice [12]–[14]. X-linked mental retardation is associated in a few patients with loss of expression of *ZDHHC15* [15] and in others with frameshifts, splice or missense mutations of *ZDHHC9* [16]. Recently, the *Drosophila* ortholog of *Zdhhc8* (*App*) was shown to play a key role in patterning and growth control of imaginal discs [17]. However, very little is known about specific palmitoylation functions during normal mammalian development.

Several lineage-restricted stem cell populations exist in the adult skin and contribute to renewal of only their own specific niche under normal steady-state conditions [18]. Their progeny proliferate, migrate and terminally differentiate along the lineages of the interfollicular epidermis (IFE), hair follicle and sebaceous gland [reviewed in 19]. The cornified layer of postnatal skin is constantly shed and replenished by progeny of the epidermal stem cells in the basal IFE, which proliferate, differentiate and migrate suprabasally. Similarly, hair shafts are shed and replaced in a cycle of regression (catagen), rest (telogen) and regeneration (anagen). During each anagen, stem cells, residing in the permanent bulge region, are mobilized to provide hair follicle progenitors, which differentiate into eight different lineages that make up the hair shaft (consisting of the medulla, cortex and hair shaft cuticle), the inner root sheath (IRS) (consisting of the inner root sheath cuticle, Huxley's and Henle's layers), the companion layer cells and the outer root sheath (ORS). Also within the permanent portion of the follicle is the sebaceous gland which produces lipid-rich sebum to lubricate the skin and hair, in addition to providing antibacterial activity. Sebum is released by disintegrating sebocytes that are continuously replaced from progenitors in the periphery of the gland. These three stem cell lineages require a precise balance of self-renewal and differentiation of their committed progeny. However under certain experimental conditions or genetic manipulations, stem cells from one niche can contribute to hair, IFE and sebaceous gland lineages [20],[21], highlighting the interdependence of these epidermal compartments in maintaining homeostasis.

The depilated mutation (*dep*, MGI:94884) results in a recessive phenotype characterized by variable hair loss, with thinner and shorter hairs remaining in a greasy coat. Recombination experiments show that the phenotype is due to a defect in the epidermis, rather then the dermis [22]. Here, we genetically map and further characterize the *dep* mutant and show that it carries a single amino acid deletion in Zdhhc21, resulting in loss of PAT activity. A detailed study of the phenotype demonstrates that lack of palmitoylation by Zdhhc21 results in hyperplasia of the IFE and sebaceous glands and delayed differentiation of the hair shaft. Furthermore, we identify Fyn, a member of the Src family of tyrosine protein kinases required for keratinocyte differentiation, as a direct palmitoylation target of Zdhhc21 and demonstrate its mislocalization within *dep* mutant follicles.

## Results/Discussion

### Mutation in Zdhhc21 causes the dep phenotype

**Figure 1:**
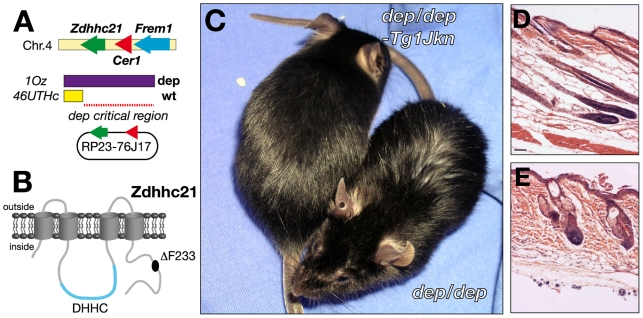
Identification and transgenic rescue of the *dep* mutation. (A) Mapping the dep interval against the b-del complex. When *dep* is crossed with the b-IOZ deletion mutant (purple), offspring exhibit the hairloss phenotype. When crossed with the b-46UTHc deletion mutant (yellow), the hairloss phenotype disappears, indicating that the *dep* mutation lies within the genomic interval between the distal breakpoints of the 2 deletions. (B) Schematic of Zdhhc21 protein with *dep* C-terminal 3bp deletion resulting in the loss of a single highly conserved residue, phenylalanine (F) at position 233. The cysteine-rich domain containing a conserved DHHC motif is shown in blue on the cytoplasmic side. (C) The BAC clone RP23-76J17, which harbors the intact genomic sequences of *Zdhhc21* and *Cer1* successfully rescues the *dep* phenotype, shown at 6.5 weeks. (D) Transgenic *dep* mutant skin appears histologically normal and correct timing hair follicle differentiation is also restored. (E) Non-transgenic mutant littermate control.

The location of the *dep* mutation has previously been defined by complementation against a set of chromosomes bearing deletions centred on the *Tyrp1* gene [23]. The endpoints of those deletions defining the proximal and distal boundaries of the candidate interval were further refined using polymorphic markers on mice carrying the deletion chromosome opposite a *Mus spretus* chromosome [24],[25, data not shown]. The candidate location of *dep*, defined by the deletions 46UThc proximally and 1OZ distally, is only 160kb in length and contains all or part of just 3 genes: *Zdhhc21*, *Cer1* and *Frem1* (Figure 1A). Two of these have existing established mutations.

*Frem1* is associated with 2 ENU-induced alleles and the classical mutation *head blebs* (*heb*) [26] which result in an embryonic blebbing phenotype, and is a mouse model for Frasers Syndrome. Furthermore, a genetic complementation analysis between a *Frem1* mutant (*bfd*) and *dep* produces normal mice (personal communication, Monica Justice), indicating *Frem1* is not allelic to *dep*. There are several knockout mutant alleles of *Cer1* but none of these exhibit the *dep* phenotype [27]–[29]. We have sequenced all known exons of both *Frem1* and *Cer1* in *dep* DNA have found no mutations. Additionally no non-coding RNAs are annotated or predicted within this interval (miRBase: microrna.sanger.ac.uk, Ensembl: www.ensembl.org, VEGA: vega.sanger.ac.uk).

However, sequencing of the 7 exons of *Zdhhc21* (MGI:1915518) in *dep* mutants revealed a 3-bp deletion which results in the deletion of a single, highly conserved, phenylalanine residue (del-233F) close to the C terminus of the protein (Figure 1B).

Although this deletion was the only coding alteration found in the candidate interval, it remained possible that an undetected non-coding mutation could affect expression of genes outside the interval. To establish the causative link between *Zdhhc21* and the *dep* phenotype, we generated transgenic mice containing the bacterial artificial chromosome, RP23-76J17, containing only *Zdhhc21* and *Cer1* (Figure 1A). When crossed onto a *dep* background, this transgene rescues the hair phenotype to a smooth and shiny dorsal coat, indistinguishable from wild-type, whilst the hair of nontransgenic littermates retains the greasy and disorderly *dep* phenotype (Figure 1C). Later in life, non-transgenic mutant littermates lose their hair, whilst the transgenic mice do not. Skin sections of transgenic rescued mice show a normal histological appearance, confirming that the *dep* phenotype is fully rescued (Figure 1D and 1E).

### Zdhhc21-del233F is mislocalised and lacks PAT function

Zdhhc21 has previously been demonstrated to have palmitoyl transferase (PAT) activity. Among 23 Zdhhc members tested, endothelial nitric oxide synthase (eNOS, Nos3) [30] and lymphocyte-specific protein tyrosine kinase (Lck) [31] were found to be robustly palmitoylated by Zdhhc21. Using these substrates, we examined whether the *dep* mutant Zdhhc21 protein retains PAT function.

**Figure 2:**
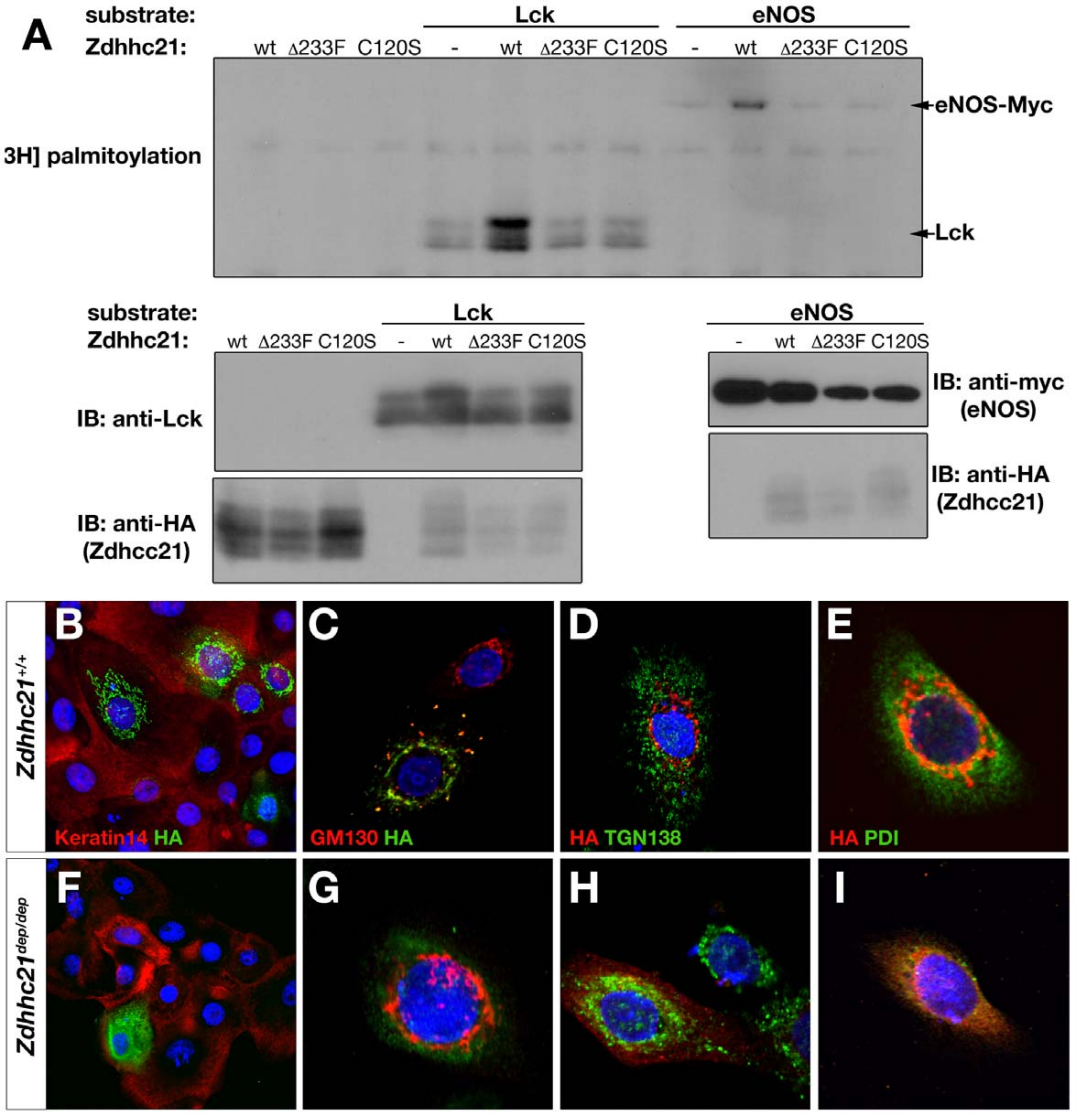
*dep* mutation disrupts PAT activity and localization of Zdhhc2*1*. (A) *[^3^H]*Palmitate fluorography of individual *Z*dhhc21 (wild type, *dep* and C120S) HA-tagged constructs co-transfected with eNOS or Lck into HEK293 cells. Increased incorporation of [^3^H]palmitate into targets is observed with the wild type construct. Neither mutant shows palmitoylation activity above background. Immunoblots using anti-HA (Zdhhc21 constructs), anti-myc (eNOS) and anti-Lck control for loading. (B–I) Immunofluorescence of primary keratinocytes transfected with wild type (B–E) and *dep* (F–I) HA-tagged Zdhhc21 cDNAs. (B,F) Epidermal marker Keratin 14 (red) and anti-HA (green) antibody staining. While wild type protein shows discrete and compartmentalized localization, the mutant protein is diffuse. (C,G) cis-Golgi network marker GM130 (red) and anti-HA (green) antibody staining. (D,H) trans-Golgi marker Tgn138 (green) and anti-HA (red). (E,I) ER marker PDI (green) and anti-HA (red).

To test PAT activity, plasmids encoding tagged wild-type and mutant Zdhhc21 proteins were cotransfected with plasmids expressing *Lck* or eNOS (*Nos3*). Palmitoylation of substrates was assessed by metabolic labeling with [^3^H]palmitate followed by SDS-PAGE and fluorography [8],[9]. Wild-type Zdhhc21 protein enhanced both eNOS and Lck palmitoylation, whilst the del233F protein showed no enhancement over background palmitoylation. A second mutant protein, C120S, in which the cysteine residue in the conserved DHHC motif was mutated, was also inactive in this assay (Figure 2A).

As mislocalisation of the mutant protein could affect its function *in vivo*, we examined the cellular localization of tagged variants of Zdhhc21 proteins in cell culture. In primary keratinocytes, HA-tagged wild type Zdhhc21 localizes to highly specific cytoplasmic structures, which co-localise with the cis-Golgi marker GM130, consistent with previous studies showing localization of other Zdhhc proteins to the Golgi (Figure 2C) [8],[30]. In contrast, Zdhhc21-del233F colocalizes with the endoplasmic reticulum (ER) marker, protein disulfide isomerase (PDI), demonstrating that *dep* mutant protein is unable to target specifically to the Golgi and appears to be trapped in the ER. (Figure 2I). We further verified these observations by transfection in NIH-3T3 cells, and demonstrated the mislocalisation and lack of PAT activity of additional mutant forms of the protein (Figure S1)

### Zdhhc21 is a PAT expressed in epithelial tissues

To define the target tissue in which PAT function is required for normal hair development, *Zdhhc21* mRNA and protein expression were analyzed at embryonic and postnatal time-points related to hair follicle morphogenesis and cycling.

In the developing skin, Zdhhc21 expression could not be detected prior to hair follicle induction (E13.5) or early morphogenesis (E14.5) (data not shown). Expression of Zdhhc21 is initially detected in the inner root sheath (IRS) of developing vibrissae follicles at E16.5 (Figure S2) and later in the developing IRS of E18.5 pelage follicles (data not shown).

**Figure 3:**
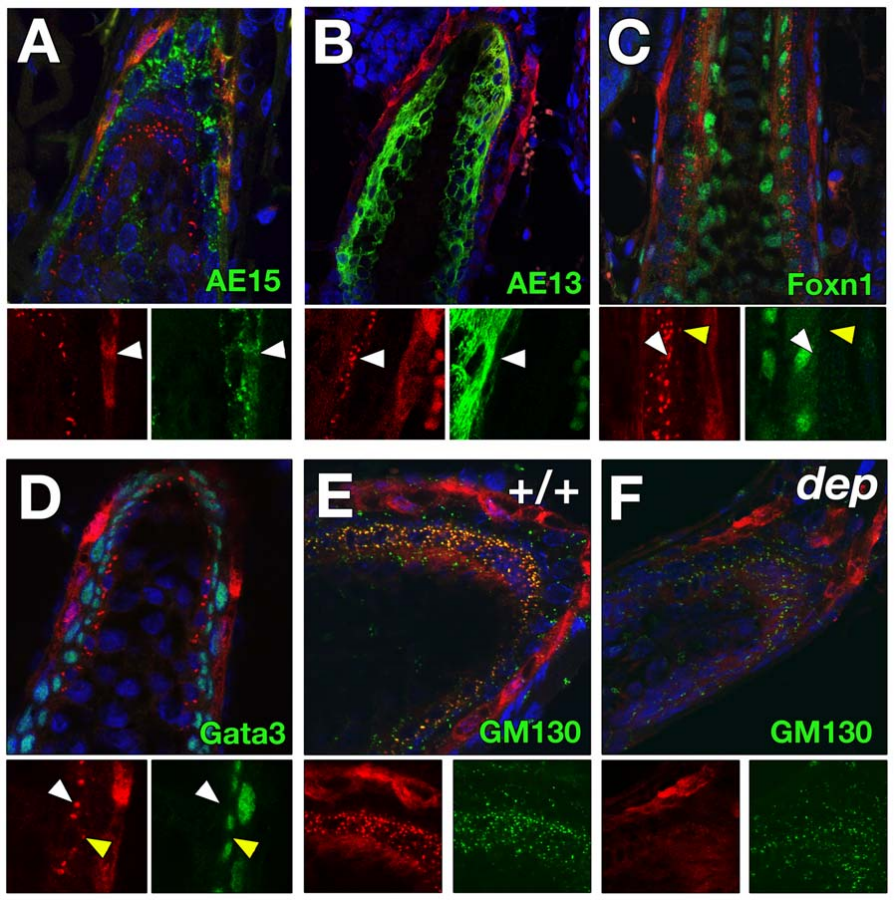
Zdhhc21 expression is restricted to differentiated post-mitotic lineages of hair follicles. Confocal slices (1µm) of Zdhhc21 protein expression in wild type (A–E) and *dep* (F) P28 anagen follicles. (A) Ubiquitous Zdhhc21 (red) expression colocalizes with the outermost layer of AE15 (green) expression. (B) Punctate Zdhhc21 staining (red) colocalizes with the outermost layer of AE13 expression (green: weaker than inner cortex expression) and (C) Foxn1 expression (white arrowhead). (D) Single foci of Zdhhc21 staining (red: yellow arrowhead) per cell of the IRS cuticle is seen in the innermost Gata3-positive layer (green). (E) In wild type skin, punctae of Zdhhc21 (red) colocalize with GM130 (green), whilst diffuse staining is observed in dep mutants (F). Arrowheads mark same region in single channels.

Postnatally, Zdhhc21 exhibits two patterns of expression in distinct layers of more distal post-mitotic lineages in the hair bulb. Strong ubiquitous cellular expression of Zdhhc21 is detected in a single layer of the IRS (Figure 3A). Double immunofluorescence with antibodies against trichohyalin (AE15) (Figure 3A) or Gata3, which is expressed only in Huxley's layer and the IRS cuticle (Figure 3D), demonstrated partial co-localization with trichohyalin but not Gata3, indicating Zdhhc21 is expressed in Henle's layer, the outermost IRS layer. A second Zdhhc21 expression domain, marked by punctate staining, is found predominantly in the outermost layer of cells expressing hair cortex keratins (AE13-positive) (Figure 3B, white arrowhead) and Foxn1-positive cells (Figure 3C, white arrowhead), indicative of the hair shaft cuticle. A less prominent but similarly punctate pattern is found in the adjacent Gata3-positive IRS cuticle cells (Figure 3C and 3D, yellow arrowhead). As in cell culture, these Zdhhc21-positive punctae colocalize with cis-Golgi marker GM130 *in vivo* suggesting that the protein in these cells is active in palmitoylation (Figure 3E). Importantly, while *Zdhhc21* transcript expression is not altered in *dep* follicles (Figure S2D and S2E), mutant Zdhhc21 protein is mislocalized in both cuticle lineages where it shows diffuse staining (Figure 3F, Figure S3). Together, the loss of *in vivo* Golgi localization of Zdhhc21 in *dep* mutants and the resulting mutant hair shaft phenotype suggest that Zdhhc21 function is primarily required in the cuticle layer. Both patterns of hair follicle expression are hair cycle dependent; expression of Zdhhc21 cannot be detected in telogen (Figure S2J) or very early anagen follicles, but it is first expressed in nested layers of the IRS and cuticle of anagen and catagen follicles (Figure S2). Comparable cyclic expression of Zdhhc21 during this postnatal hair cycle is also observed in *dep* mutant skin. Notably, the onset of expression in differentiating lineages in the anagen follicles correlates with the first sign of abnormal morphology (Figure S3).

Outside the cycling portion of the hair follicle, we find specific cellular Zdhhc21 protein strongly present in the degenerated remains of the IRS surrounding the isthmus, in the permanent portion of the follicle (Figure S2I). Importantly, expression of Zdhhc21 mRNA or protein cannot be detected in the bulge, IFE or in the sebaceous gland at any stage of the hair cycle.

### Zdhhc21 is required for epithelial homeostasis

The *dep* phenotype can be identified macroscopically within the first postnatal week as a greasy and disorderly hair distribution, as previously reported [22]. To determine the cellular basis of the observed abnormalities, we conducted histological and molecular analyses of skin samples at a range of developmental stages.

**Figure 4:**
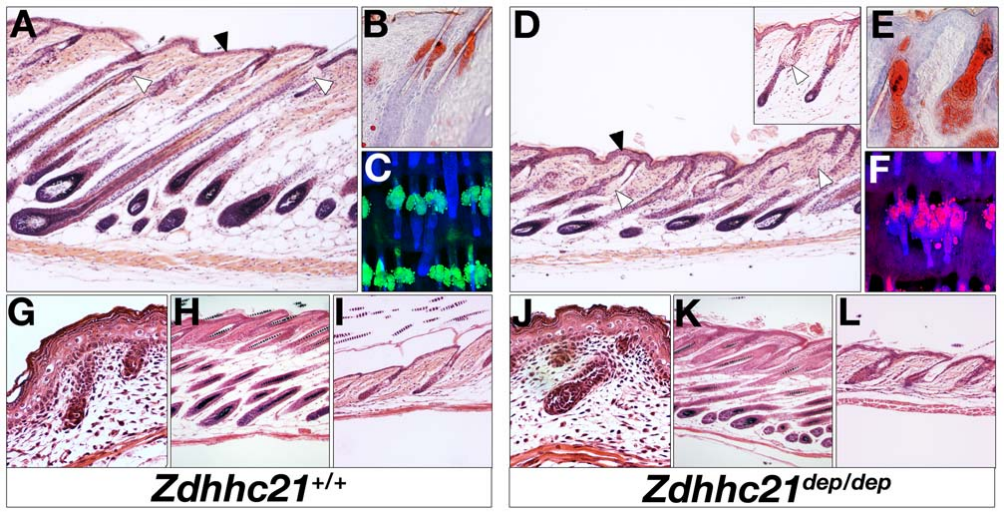
Zdhhc21 is required for epidermal differentiation and patterning. *dep* mutant skin displays pronounced defects postnatally associated with anagen stages of hair cycle including abnormal hair follicle differentiation, and interfollicular and sebaceous hyperplasia. Stages shown: anagen P28 (A–F), late embryonic morphogenesis E18.5 (G,J), early catagen P14 (H,K) and telogen P21 (I,L). Hematoxylin and eosin staining (A,D,G–L). Oil-Red-O staining of cryosections (B,E). Nile red wholemount staining of P28 tail skin(C,F). Insert in (A) shows histology of a “hairless” dorsal region at P28: note the more severe follicular phenotype. Filled arrowheads mark the interfollicular epidermis and open arrowheads point to examples of sebaceous glands.

Dorsal skin from *dep* embryos at E14.5 and E18.5 have follicle morphology and numbers comparable to wild type (Figure 4G and 4J and data not shown), indicating that Zdhhc21 function is dispensable for hair follicle patterning and morphogenesis. The first abnormalities in *dep* mice are observed shortly after birth where mild sebaceous gland hyperplasia and slight thickening of the IFE develop at P5. While *dep* mutants appear to progress through the first hair cycle normally (Figure 4H and 4K), by telogen, defects in the permanent portions of *dep* skin are apparent and include thickening of the IFE and a dilated infundibulum (Figure 4I and 4L). By the onset of the second hair cycle around P28, the dep follicles are growth retarded and immature compared to littermates (Figure 4A and 4D) coincident with the onset of Zdhhc21 expression (Figure S3). In addition, the thickening of IFE and sebaceous gland hyperplasia appear more prominent (Figure 4D, arrowed). Staining for lipids reveals enlarged sebaceous glands with an excess of sebum (Figure 4E and 4F), underlying the greasy appearance of the coat at this stage. In some *dep* animals, from P28 onwards, small epidermal cysts containing keratinized material can be observed in the upper portion of the dermis (not shown). Given the hyperplastic changes observed in the upper portions of *dep* follicles, we asked whether the closely associate bulge stem cell niche was also perturbed. Keratin 15 (K15) is a marker for these cells, and indeed, the K15-positive population is expanded in *dep* mutants, although its expression remains restricted to the bulge niche, suggesting that changes in the size and shape of the *dep* bulge during the hair cycle could impact progenitor allocation to various epidermal compartments (Figure S5F and S5L, data not shown).

The hyperplastic phenotype of *dep* IFE and sebaceous glands is most prominent during anagen in younger skin, when growth stages of the hair cycle are highly synchronized. To determine whether this hyperplastic phenotype was due to increased proliferation of these non-follicular compartments, we carried out BrdU pulse labeling cohorts of P32 gender-matched animals. These studies revealed a small but significant increase in the fraction of BrdU positive *dep* IFE cells (8.314±1.493, n = 2, p<0.005) compared with heterozygous (6.790±1.8223, n = 2) or wild type controls (6.686±1.711, n = 2) (Figure S4L). A greater increase in percentage BrdU positive cells was observed in *dep* sebaceous glands (11.46±2.784, n = 2, p<0.05) compared to controls (heterozygous: 6.881±2.499; wild type: 7.882±2.868). A concomitant decrease of BrdU labeling is observed in *dep* mutant follicles during anagen (Figure S4K). Additional proliferation markers, including the M-phase marker phospho-histone H3 and a general marker Ki67 identifying all phases of the cell cycle, confirm this change in proliferation is relatively small and is restricted to the basal compartment (Figure S4A, S4B, S4C, S4D, S4E, S4F, S4G, S4H, S4I, S4J).

**Figure 5:**
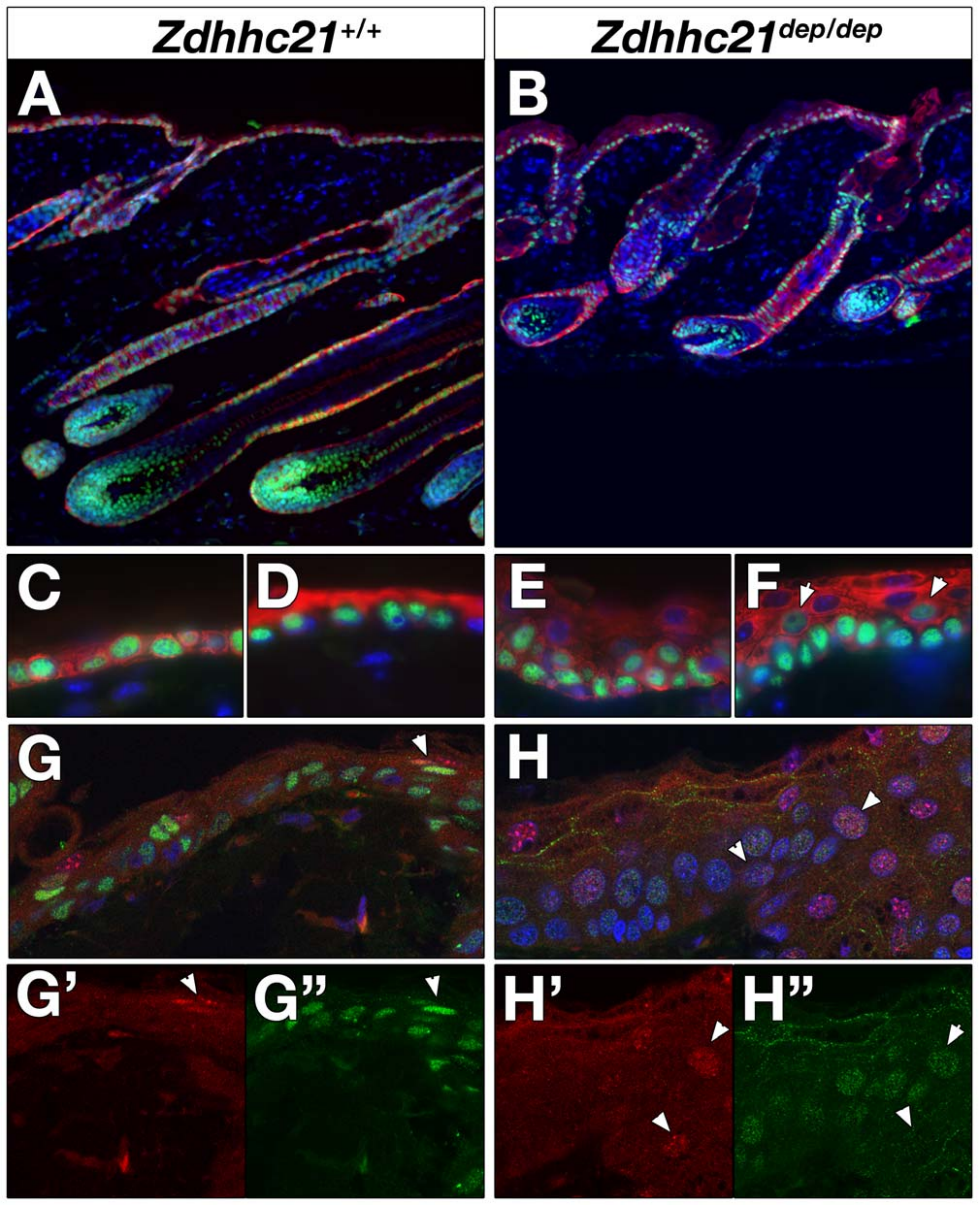
Loss of Zdhhc21 disrupts balance between proliferation and differentiation in anagen interfollicular epidermis. P28 wild type (A,A′,C,D,G,H,K,L) and *dep* (B,B′,E,F,I,J,M,N) skin. *dep* mutants show expansion of K5+ (red), p63+ (green) progenitor compartment (A–D). p63+ progenitors (green) are expanded into terminally differentiating K10+ (red) layers of *dep* IFE (D,F). Expression of transcriptional regulator of differentiation Gata3 in the basal and suprabasal keratinocytes is greatly reduced or absent in *dep* IFE with a parallel increase in proliferative basal phospho-ERK signals (green; Gata3, red; phospho-p42/44, G,H). (G–H″) single channels of region of interest in merge shown in (G,H).

We asked whether aberrant terminal differentiation of keratinocytes in the IFE also contributes to the *dep* phenotype, such that an expanded progenitor pool contributing to the IFE could result in an increase in cell number in the stratified layers. Immunolabelling of basal cell markers p63 and keratin 5 (K5) showed an expansion of this progenitor compartment (Figure 5A, 5B, 5C, and 5E). Furthermore, p63-positive cells were found in thickened K10-positive spinous layer in *dep* skin (Figure 5D and 5F, arrowed). The terminal differentiation markers loricrin and filaggrin were only slightly expanded in *dep* mutants (Figure S5A, S5B, S5G, S5H) indicating that differentiation in *dep* mutants occured but was significantly delayed. Interestingly, the transcription factor Gata3, which is normally expressed in the basal and suprabasal layers of the IFE where it directs keratinocyte and lipid based barrier differentiation programs [32],[33], is strongly reduced in *dep* IFE (Figure 5G and 5H, arrowed) consistent with the observed delay in differentiation. Reduced levels of Gata3 in the *dep* IFE during anagen may contribute to defects in lipid biosynthesis required for barrier function, which may give rise indirectly to the hyperproliferative phenotype observed [34]. However, unlike *Gata3* knock-out skin [32], delays in the establishment of embryonic barrier function by dye penetration assays were not seen and keratinocyte terminal differentiation program in embryonic skin occurred normally (Figure S6). Furthermore, phenotypes associated with impaired barrier function, including failure to thrive or red shiny skin, were not observed in *dep* neonates. These observations suggest that any barrier defects present in *dep* mutants are likely quite subtle and limited to a postnatal window. In contrast to the decrease in Gata3 and altered terminal differentiation, an increase in phospho-ERK staining, indicative of growth factor and integrin signaling linked to increased proliferation in the basal layer of the IFE [35], is observed in *dep* mutants (Figure 5G and 5H, Figure S5D, S5D′, S5D″, S5E, S5E′, S5J, S5J′, S5J″, S5K, S5K′, arrowed). These observations together suggest that the thickening of the IFE observed during anagen in *dep* mutants is due to continued division and delayed differentiation of the expanded basal progenitor compartment after leaving the basement membrane.

The restricted expression of Zdhhc21 in the IRS and cuticle of the hair follicle is hard to reconcile with a direct effect on proliferation and differentiation in the IFE and sebaceous gland. One possibility is that the physiologically relevant palmitoylation targets are highly diffusible signals, or are regulators of such signals. Alternatively, Zdhhc21 may act locally in the follicle to indirectly impact non-follicular lineages as a consequence of hair abnormalities in *dep* mice. Such a phenomenon is seen in *K14-Cre*-induced knockout of the hair-follicle specific,transcription factor *Dlx3*, where the resultant abnormal and undifferentiated hair shafts are accompanied by hyperplastic sebaceous glands [36].

### Hair cycle signal transduction defects in *dep* mutants

**Figure 6:**
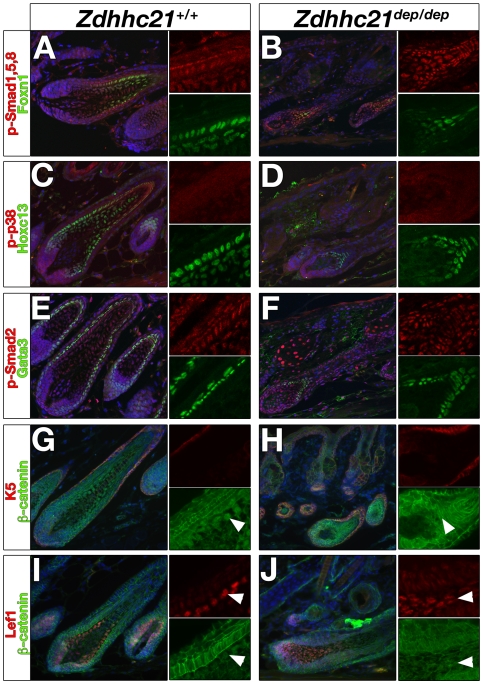
Anagen signalling is delayed in *dep* follicles. Wild type (A,C,E,G,I) and *dep* (B,D,F,H,J) P28 dorsal follicles. Several key signalling pathways required for postnatal hair development are not affected in *dep* mutants. Canonical BMP signalling (red; phospho-Smad-1/-5/-8, A,B), non-canonical BMP/TGF-β signalling (red; phospho-p38, C,D) and canonical TGF-β signalling (red: phospho-Smad-2/-3 E,F) are expressed in delayed *dep* follicles. Expression and nuclear localization of downstream canonical Wnt effectors β-catenin (green; β-catenin, G–J) and Lef-1 (red; Lef-1, I,J) are reduced in *dep* hair matrix and shaft (arrowed). Of the transcription factors regulating differentiation of anagen hair follicle lineages, expression of Wnt transcriptional target Foxn1 is reduced or absent in *dep* follicles (green; Foxn1 A,B), while Hoxc13 is expressed in all *dep* follicles (green; Hoxc13 C, D). Gata3 is expressed in all *dep* follicles although levels of expression may be slightly reduced. (green; Gata3 E,F). Along side merge panel are shown single channel of regions of interest to highlight staining.

As palmitoylation is usually involved in the regulation of dynamic processes, we investigated whether key signalling events throughout the postnatal hair cycle were affected in *dep* mutant skin. Bone morphogenetic protein (BMP) signalling is required for embryonic hair follicle development and postnatal hair cycling [37]. Furthermore, conditional epidermal ablation of receptor BMPR1a [38]–[40] result in a hair loss phenotype associated with poorly differentiated hair follicles, and thickened IFE. However, no difference in expression of activated phospho-Smad1/5/8, mediators of canonical BMP signalling, was detected in mutant skin at various stages of the hair cycle (Figure 6A and 6B, data not shown). Transforming growth factor beta (TGF-β) signalling also plays a key role in hair follicle development and cycling, as well as keratinocyte differentiation [41],[42]. No difference in expression of activated canonical intracellular mediator phospho-Smad2 was observed in *dep* mutant follicles or IFE (Figure 6E and 6F, data not shown). Recent studies have suggested a key role for palmitoylation in BMP- [43] or TGF- mediated signalling events [44] via the non-canonical p38 MAPK arm; however, no alterations in phospho-p38 staining could be detected in *dep* mutants (Figure 6C and 6D). These results suggest that despite the profound follicular phenotype of *dep* mutant mice, these key developmental signals required for adult hair cycle are not broadly affected.

The range of phenotypes seen in *dep* animals is reminiscent of a reduction of Wnt signalling, which plays many important roles during hair development. Precise levels of β-catenin activation are required for differentiation into specific epidermal lineages. High levels of β-catenin signaling promote hair follicle formation [45],[46] and normal differentiation of the hair shaft [47]. Low levels of Wnt/β-catenin signaling promote terminal differentiation of the IFE and sebaceous glands [48],[49]. To determine whether a reduction in Wnt signalling is seen in *dep* mutant skin, we analyzed Wnt responses in embryonic and adult skin by immunohistochemistry. Wnt responses during embryonic hair follicle morphogenesis appear normal in *dep* embryos (data not shown). At the initiation of the first, synchronized, anagen phase (P24), prior to expression of Zdhhc21, both control and *dep* littermates show nuclear Lef1 in the dermal papilla and surrounding secondary hair germ (Figure S7A, S7B, S7C, S7D). However, in *dep* mice, propagation of this anagen response appears defective and differentiation of the hair shaft and cortex is significantly delayed. By P28, at the onset of Zdhhc21 expression when wild type hair is well established in anagen, the delayed *dep* hair follicles fail to expand strong Lef1 and nuclear β-catenin expression in the matrix and precortex (Figure 6H and 6J). Accordingly, the Lef1 transcriptional target, *Foxn1*, which regulates expression of hair specific keratins, is strongly reduced or absent from mutant follicles (Figure 6A and 6B, Figure S7I and S7K) as are acidic hair shaft keratins (AE13), (Figure S7J and S7L) consistent with the delayed state of development. By contrast expression of homeodomain transcription factor Hoxc13, which also regulates expression of several hair shaft keratins, is still detected in all *dep* mutant follicles at this stage of anagen (Figure 6C and 6D). Surprisingly, unlike the profound reduction in Gata3 expression observed in the *dep* IFE and similarities between follicular phenotype observed in conditional *Gata3* mutant mice [50], Gata3 is still detected in all *dep* follicles although at slightly reduced levels throughout anagen (Figure 6E and 6F, Figure S3). By P35, many *dep* follicles express levels of Lef1 and hair-shaft keratins comparable to controls, although the morphology of *dep* follicles remain misshapen and misoriented (Figure S7M, S7N, S7O, S7P). Interestingly, some regions in *dep* mice continue to remain visibly “hairless”, although histological analysis reveals normal numbers of retarded follicles which fail to proceed through anagen and form functional hairs (Figure 4D, insert).

Our data suggest that a number of signalling pathways required for epidermal homeostasis are disrupted in the absence of Zdhhc21 PAT activity. During the anagen phase of the hair cycle there is a reduction of Wnt responses in the hypoproliferative *dep* follicles and an increase in phospho-ERK signalling in the hyperplastic mutant IFE. Which of these phenotypes are direct or indirect consequences of the loss of Zdhhc21 palmitoylation remains to be addressed. Given we cannot detect Zdhhc21 expression outside the follicle, we suggest the follicular phenotype observed in *dep* mutants is the primary cause where defects in hair shaft differentiation during anagen perturb processes at a distance in the IFE and sebaceous glands. Importantly, palmitoylation may influence the quality and the quantity of a signalling event rather than acting as an absolute ON/OFF switch. This is in keeping with the observation that the amplification of Wnt responses during early anagen is very delayed, and not completely blocked, suggesting some threshold could be operating and is eventually met in mutant follicles.

### *In vivo* palmitoylation targets for Zdhhc21

At the synapse, palmitoylation mediates dynamic changes in membrane associations of pools of target proteins involved in signaling, cell adhesion and trafficking [51]. Given the rapid remodeling observed in the hair cycle, it is tempting to speculate that similar processes are involved in the skin and to ask what are the biologically relevant targets of palmitoylation. It should be noted, that while several targets have been identified for each of the 23 Zdhhc PATs, each of these target so far is palmitoylated by multiple PATs, at least *in vitro*. This suggests a level of functional redundancy in the palmitoylation machinery exists. It also suggests that the *dep* phenotype could result from the loss of palmitoylation of one or more targets.

We reasoned that any direct target of PAT activity must be expressed in the same cells in which we detect Zdhhc21 expression. One possibility is the known Zdhhc21 target, eNOS [30], which is expressed in the skin [52]. However, observation of *eNOS* mutant mice indicates that this is not required for normal skin and hair development [53, data not shown], suggesting it is unlikely to be the key palmitoylation target of Zdhhc21 in skin.

Given that Zdhhc21 expression is restricted to hair follicles but multiple epidermal lineages are affected in *dep* mutants, we asked whether diffusible Wnt proteins could be functional palmitoylation targets for Zdhhc21. Wnt proteins are known to be palmitoylated, and this modification is essential for their function [54]. However, this is believed to be mediated by the ER protein porcupine (PORC), a PAT unrelated to the Zdhhc family [55]. Nevertheless, we tested three candidate Wnts (Wnt3a, 5a and 10a), which are expressed in domains that overlap with Zdhhc21 expression [56]. Although these Wnts are predicted to have multiple palmitoylation sites (CSS-Palm, data not shown) [57], none are directly palmitoylated by Zdhhc21 (data not shown). Trafficking of Wnt ligands from the Golgi to endosomes requires the cargo receptor Wntless/Evi (Wls), a seven-transmembrane protein expressed in the Golgi [58]. Sustained Wnt signaling also requires that this cargo receptor be recycled via the retromer complex. Similar cargo proteins have been shown to require DHHC-dependent palmitoylation for retrograde sorting [59]. However, no palmitoylation of Wls by Zdhhc21 was detected in our co-transfection assay (data not shown). While it remains possible that Zdhhc21 acts locally in a subset of Wnt-responding cells in the hair follicle required for proper hair shaft differentiation (*i.e.* through modulation of receptor complexes or intracellular signal transduction), we have been unable to establish a direct link between palmitoylation and Wnt responses in this present study.

**Figure 7:**
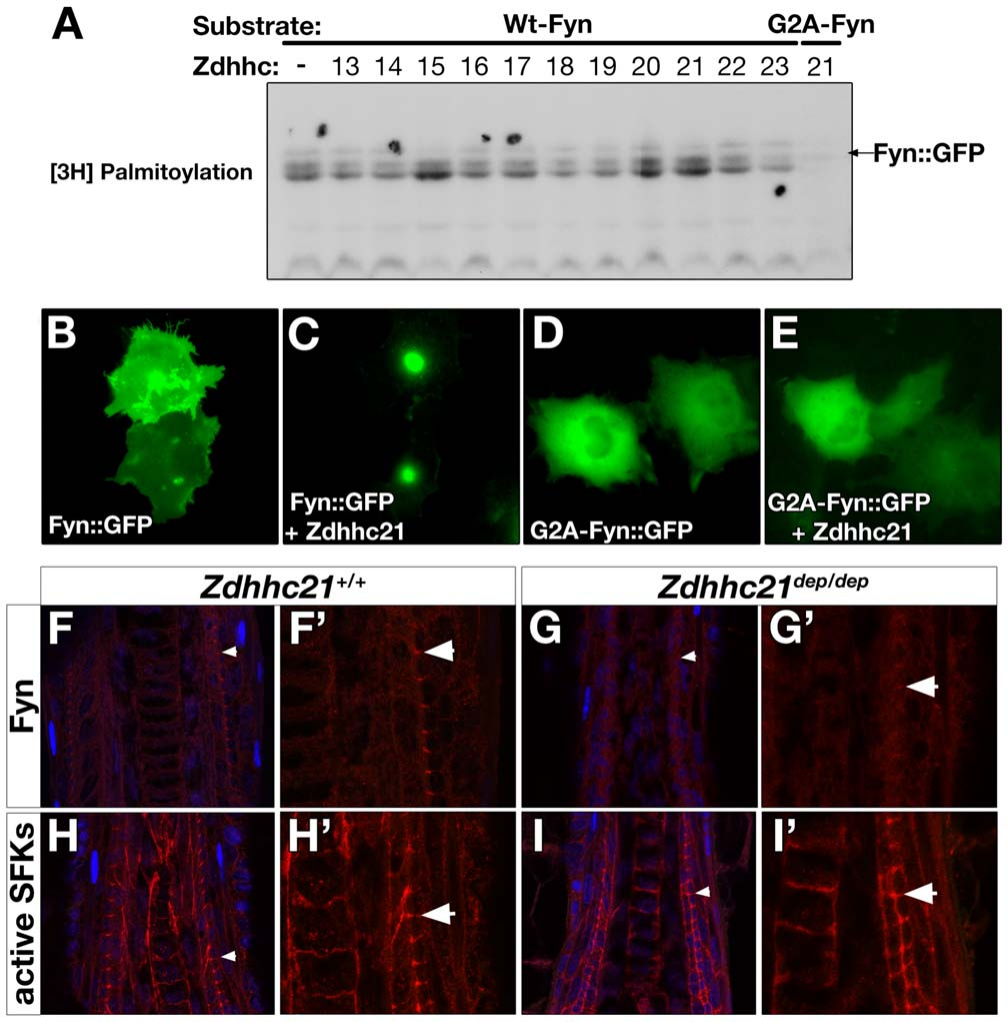
Zdhhc21 palmitoylation of Fyn is required for localization *in vitro* and *in vivo*. (A) [3H]Palmitate fluorography of subset of panel of all mammalian Zdhhc HA-tagged constructs co-transfected with Fyn::GFP into HEK293 cells. Increased incorporation of [3H]palmitate into targets is observed with Zdhhc15, −20 and −21. A myristoylation-deficient Fyn mutant is not palmitoylated following cotransfection with Zdhhc21 (G2A, final lane). (B–E) Immunofluorescence of HEK293 cotransfected with Fyn::GFP alone (B), Fyn::GFP and wild type HA-tagged Zdhhc21 cDNA (C), G2A mutant Fyn::GFP alone (D) and G2A mutant Fyn::GFP and HA-tagged Zdhhc21 (E). Zdhhc21 causes increased perinuclear accumulation of Fyn::GFP, a domain associated with localization of other palmitoylated targets including PSD-95 and GAP-43. This localization is not observed with G2A mutant Fyn::GFP (D). (F–I) Confocal images (0.5µm) of immunostaining of Fyn (red: F,G) and active SFKs (red: H–I) in P32 anagen dorsal hair follicles in wild type (F,H) and dep mutants (G,I). Arrowheads mark region of interest in IRS cuticle that are enlarged in single channel images (F′–I′) where Fyn localization to membranes between IRS cuticle cells is lost in dep mutants.

While the Src family kinase, Lck, is a known target of Zdhhc21, it is not required for keratinocyte differentiation nor do *Lck* mutants have any gross skin phenotype [60]. We therefore considered whether other related kinases that are epidermally expressed could be potential palmitoylation targets. Fyn is indeed expressed in the skin where it plays a role in keratinocyte differentiation *in vitro* and *in vivo* [60], in part through down-regulating EGFR signaling [61]. The role for Fyn in hair follicle development and cycling remains unclear. Aged *Fyn* ^−/−^ *Fak^+/−^* mice develop progressive hair loss with IFE and sebaceous gland hyperplasia, but this is not observed in *Fyn* ^−/−^ mutants [62]. We therefore tested GFP-tagged Fyn with a panel of Zdhhc PATs by co-transfection and metabolic labelling. Fyn is palmitoylated in our *in vitro* assay by Zdhhc2, 3, 7, 10, 15, 20 and 21 (Figure 7A, data not shown). Palmitoylation of Fyn by Zdhhc21 results in efficient targeting of Fyn to the perinuclear region in HEK cells (Figure 7B and 7C). Fyn is also subject to myristoylation, an irreversible covalent lipid modification involved in membrane targeting and signaling. Interestingly, we show a mutant Fyn construct lacking the myristoylation site (Fyn-G2A) cannot be palmitoylated by Zdhhc21 or correctly targeted (Figure 7D and 7E), suggesting palmitoylation of Fyn is downstream of the myristoylation event.

To test whether Fyn was a viable *in vivo* target of Zdhhc21, we examined localization Fyn in wild type and *dep* follicles. In wild type anagen (P32) follicles, Fyn is initially expressed diffusely in the hair bulb becoming very discretely localized to membranes at junctions between cells of the IRS cuticle with differentiation (Figure 7F and 7F′, arrowed). This localization is weaker and more diffuse in *dep* follicles (Figure 7G and 7G′, Figure S8A and S8B). These results were confirmed using an antibody which recognizes the active, phosphorylated forms of all Src-family kinases (SFKs) in addition to Fyn. The active SFKs show a broader expression pattern with striking membrane localization, including the junctions between cells of the IRS cuticle (Figure 7H, Figure S8C). In contrast, while general expression of active SFKs is not altered in *dep* mutant follicles, uniform active SFK is seen around cells of the IRS cuticle (Figure 7I, Figure S8D). Given that the Zdhhc21 Golgi-localization observed in the IRS cuticle of wild type follicles is lost in *dep* mutants and that Golgi-localization is dependent on auto-palmitoylation via the PAT activity of wild type Zdhhc21, our results suggest that Zdhhc21-mediated palmitoylation of Fyn is required *in vivo* for Fyn's discrete localization in the differentiating IRS cuticle. It is interesting to note that in the despite the proliferation and differentiation defects observed in the *dep* mutant IFE, and Fyn's established role in keratinocyte differentiation, the localization of Fyn in the *dep* IFE is normal, although it is delayed as expected given the expanded basal compartment in *dep* mutants (Figure S8E and S8F).

Our data demonstrates that Fyn is a direct palmitoyaltion target for Zdhhc21 *in vitro* and dysregulation of Fyn occurs *in vivo* in *dep* mutant follicles. In assessing the *dep* phenotype, it is worth noting that the consequences of dysregulated palmitoylation may not mirror those of gene ablation studies, as palmitoylation has the potential to modulate cell signaling in a complex manner. Furthermore, the phenotypic features of *dep* mice extend beyond those likely caused by the loss of Fyn activity, correlating with the broad substrate specificity of different Zdhhc proteins. To comprehensively tackle the functional requirement of Zdhhc21, the use of global approaches, recently applied in yeast, will be necessary to compare the palmitoylated proteome of *dep* mutant and wild type cells [63].

This study is the first to highlight a role for palmitoylation in mammalian development and homeostasis. We have demonstrated that loss of Zdhhc21 function in *dep* mutants results in defects in all three epidermal lineages, including hyperplasia of the IFE and sebaceous glands with a delay in hair follicle differentiation. Given the highly restricted pattern of Zdhhc21 expression to the differentiating hair follicle, our results demonstrate that defective palmitoylation can have far-reaching effects disrupting epidermal homeostasis by altering the balance between proliferation and differentiation. Although the full identity of direct and biologically relevant palmitoylation targets in the skin remains unknown, we show Zdhhc21 can directly palmitoylate Fyn *in vitro* and this modification affects Fyn localization both *in vitro* and *in vivo*. Future studies into the distinct and overlapping roles of additional Zdhhc members will help to fully understand the role of palmitoylation in modulating key signals during development.

## Materials and Methods

### Ethics statement

Mice were maintained in accordance with MRC Guidelines “Responsibility in the Use of Animals for Medical Research” (July 1993) and research licenced by the UK Home Office under the Animals (Scientific Procedures) Act 1986.

### Mouse husbandry and BAC transgenics

Animals were maintained in SPF environment and on a C57BL/6J background. Genomic DNA extracted from ear clips or tail biopsies was used for PCR genotyping. For *dep*, exon 7 of *Zdhhc21* was amplified by standard PCR to yield a 249bp fragment that was run on the ABI310 genetic analyzer to detect the deletion.

The *dep* genotyping primer sequences were: 5′-FAM-AGCTGACTGAAGGGCACC-3′ (Exon 7F) and 5′-AAAACCTGTAACGCATTTCCA-3′ (Exon 7R).

For transgenic rescue, purified RP23-76J17 BAC DNA (BAC PAC Resource Center (BPRC), the Children's Hospital Oakland Research Center Institute, CA) was injected into homozygous *dep* embryos. The presence of the BAC was genotyped using three markers, including CmR specific to the plasmid, as well as two markers at both ends of the BACs, amplifying the border between the BAC carrier plasmid and BAC genomic region. For timed matings for embryonic samples, the morning of vaginal plug was counted as 0.5 (E0.5). For postnatal timepoints, a set of gender-matched wild type, heterozygous and mutant littermates were aged accordingly from the day of birth 0 (P0).

### Deletion mapping

Mapping of the deletion endpoints defining *dep* was described by Smyth et al [24].

### Palmitoylation assay

cDNA encoding wild type and mutant Zdhhc21 were transfected into HEK293 cells, along with candidate palmitoylation substrates. Cells were labelled with [^3^H]palmitate for 4 hours as previously described [8],[9]. After metabolic labeling, palmitoylated proteins were analysed by SDS-PAGE. Transfection efficiency and translation of substrates was assessed by Western blotting.

### Preparation of mammalian expression plasmids and cell culture

The mammalian expression vector of wild-type Zdhhc21, pEFBos-HA-Zdhhc-21 (with EF1-alpha promoter), was provided by Masaki Fukata. It was then modified by using the QuikChange® Site-Directed Mutagenesis Kit (Stratagene), to introduce single nucleotide changes for the following Zdhhc21 alleles: L91F, C95S and C106S. For the *dep* mutation, del-233F, was modified from pEFBos-HA-Zdhhc-21 by subcloning *dep* cDNA (Access RT-PCR System, Promega) into the BamHI sites flanking the insert.

Full-length mouse Wnt cDNAs (kindly provided by Jeremy Nathans (Wnt3a), Wnt5a (Yingzi Yang) and Wnt10a (Takano Yamamoto)) were introduced into a pCMX2GFPFLAGSTOP vector (kindly provided by Nick Gilbert) to express double FLAG-tagged full-length proteins. Constructs were verified by direct DNA sequencing, using primers:

5′-CAAATGGGCGGTAGGCGTGT-3′ (Pcmxgfp2fg-seqF)

5′-TTGTCCAATTATGTCACACCA-3′ (Pcmxgfp2fg-seqR)

The Myc-tagged full length Wls cDNA used in these studies has accension number BC018381 (Catalog No: MR207034: Origene, MD).

Human foreskin keratinocytes [64] and NIH 3T3 cells (ATCC) were maintained as described. DNA plasmids were transfected into cells using Lipofectamine 2000 (Invitrogen) as per manufacturer's specifications.

### *Zdhhc21* RNA in situ probe and antibody generation

*Zdhhc21* cDNA product was generated using Access RT-PCR kit (Promega) and cloned into p-GEM-T. The RT-PCR primers used were: 5′-CATGGGCTTGATTGTCTTTGT-3′ and 5′-ACGTGATTGGCAAAGTGGTAG-3′. DIG-labeled RNA section *in situ* protocol was performed, details available on request. Custom rabbit polyclonal antibodies to Zdhhc21 were generated using a peptide comprising residues 73–87 (GRLPENPKIPHAERE+C)(Eurogentec). Pre-incubation of the Zdhhc21 antibody with its immunizing peptide blocked all signal in immunohistochemistry (Figure S2L).

### Histological and marker gene analysis

#### Tail epidermis wholemount preparations

Whole tail skin was peeled off connective tissue and bone, then incubated in 5mM EDTA/PBS for 4 hours at 37°C. Forceps were used to separate epidermis from dermis which was then fixed in 4% paraformaldehyde/PBS.

#### Alkaline phosphatase staining of dermal papilla and staging hair cycle

Paraffin embedded sections of 6µm thickness were dewaxed and rehydrated, and rinsed in PBS. Sections were incubated 15 minutes in APB (100mM NaCl, 100mM Tris pH9.5, 50mM MgCl2, 0.3% Tween-20), then either BM Purple (Roche) or Vector Red Alkaline Phosphatase kit (Vector Labs) color substrate was added. Slides were incubated at room temperature in the dark until staining developed. Sections were rinsed in water and counterstained with eosin (in the case of BM Purple), then dehydrated and mounted in DePex mounting media (BDH). Staging of hair follicles in hair cycle was done according to characteristics described in [65].

#### Oil-Red-O staining for lipids

Cryosections of P24 skin of 10µm thickness were rinsed in water, followed by 50% EtOH and stained with Oil-Red-O (60% stock in water). Slides were rinsed in 50% EtOH, followed by water and counterstained with haematoxylin. Samples were blued and washed in water and mounted in Aquamount (BDH). Oil-Red-O stock solution was prepared by dissolving 0.5g Oil-Red-O in 100ml 99% Isopropyl Alcohol.

#### Barrier function assay

Dye penetration assays were performed on late E16.5 litters as previously described [66] with the following modifications. Embryos were dissected out in cold PBS and permeabilized by taking them up and down a methanol/PBS series (25%, 50%, 75%, 100%) with 5 minute washes. Embryos were stained in 0.1% toludine blue solution for 3 minutes, then rinsed through 4 rapid PBS washes. Embryos were immediately photographed on a Nikon AZ100 macroscope using a Nikon APO 0.5× lens in PBS on 2% agarose plates.

### Immunohistochemistry and immunocytochemistry

Paraffin sections were dewaxed and rehydrated, followed by washes in TBST +0.5% Tx-100). Microwave antigen retrieval was carried out using 1mM EDTA (pH8) or citrate buffer (1.8mM citric acid, 8.2mM sodium citrate, pH6) for 20–30 minutes depending on the antigen at 900W. Cryosections samples were allowed to come to room temperature and post-fixed in acetone (−20’C for 10 minutes) followed by rinsing in water. No antigen retrieval step was required for cryosections. Slides were cooled to room temperature and washed in TBST. Slides were blocked in 10% donkey serum/TBST, followed by TBST washes. Primary antibodies were diluted in 1% donkey serum/TBST incubated on slides overnight at 4°C (Table 1). After TBST washes, secondary antibodies were diluted in 1% donkey serum/TBST and added to the slides for 60 minute incubation (Table 2). Following stringent TBST washes, nuclei were stained with DAPI (Sigma) or TOTO-3 (Molecular Probes). In the case of TOTO-3, slides were pre-incubated with RNAse A during primary antibody incubation. Slides were mounted with Vectashield (Vector) or Prolong Gold (Molecular Probes) antifade media and coverslips. Brightfield and fluorescent images were acquired using a Coolsnap HQ CCD camera (Photometrics Ltd, Tucson, AZ) Zeiss Axioplan II fluorescence microscope with Plan-neofluar objectives. Image capture and analysis were performed using in-house scripts written for IPLab Spectrum (Scanalytics Corp, Fairfax, VA). For colocalization studies, 0.5–1 µm optical slice images in Z-stacks were acquired with a Zeiss LSM510 confocal microscope and Zeiss Plan Apochromat lenses (Carl Zeiss, Welwyn Garden City, UK). LSM software was used for analysis (Carl Zeiss, Welwyn Garden City, UK).

**Table 1:** Details of primary antibodies used in this study.

Antigen	Clone Name	Host Species	Dilution	Source	Notes
AE13	N/A	Mouse	1∶10	T.T. Sun	P-IHC
AE15	N/A	Mouse	1∶2	T.T. Sun	P-IHC
β-catenin	15B8	Mouse IgG1	1∶500	Sigma	P-IHC
β-catenin	C2206	Rabbit	1∶2000	Sigma	P-IHC
β-galactosidase	CR7001RP2	Mouse IgG1	1∶1000	Cortex	P-IHC
BrDU	B44	Mouse IgG1	1∶50	BD Biosciences	P-IHC
Cleaved Caspase3 (Asp175)	9661	Rabbit	1∶400	Cell Signaling	P-IHC
Filaggrin	PRB-417P	Rabbit	1∶1000	Covance	P-IHC
Foxn1	G-20	Goat	1∶100	Santa Cruz	P-IHC
Fyn	Y303	Rabbit IgG	1∶50	Abcam	P-IHC
Gata3	HG3-31	Mouse	1∶75	Santa Cruz	P-IHC
GM130	35	Mouse IgG1	1∶200	BD Biosciences	IF, P-IHC
HA	05-904	Mouse IgG3	1∶200–400	Upstate	IF, WB
HA	HA-7	Mouse IgG1	1∶25	Sigma	IF
Hoxc13	10D4	Mouse IgG1	1∶50	abnova	P-IHC
Keratin 5	PRB-160	Rabbit	1∶1000	Covance	P-IHC
Keratin 6	PRB-169	Rabbit	1∶500	Covance	P-IHC
Keratin 10	PRB-159	Rabbit	1∶500	Covance	P-IHC
Keratin 14	AF64	Rabbit	1∶500	Covance	IF
Keratin 15	sc-56520	Mouse IgG2a	01∶50	Santa Cruz	P-IHC
Ki67	TEC-3	Rat	1∶50	DakoCytomation	P-IHC
Lck	3A5	Mouse IgG2bk	1∶600	Chemicon	WB
Lef1	N/A	Rabbit	1∶500	R. Grosschedl	P-IHC
Lef1	C12A5	Rabbit	1∶1000	Cell Signaling	P-IHC
Loricrin	PRB-145	Rabbit	1∶500	Covance	P-IHC
p63	BC4A4	Mouse IgG2a	1∶100	Abcam	P-IHC
PDI	RL90	Mouse IgG2a	1∶100	Abcam	IF
phospho-p38 (Thr180/Tyr182)	12F8 (4631)	Rabbit IgG	1∶50	Cell Signaling	P-IHC
phospho-p42/44 (Thr202/Tyr204)	E10 (9106)	Mouse IgG1	1∶400	Cell Signaling	P-IHC
phospho-p42/44 (Thr202/Tyr204)	20G11 (9106)	Rabbit IgG	1∶100	Cell Signaling	P-IHC
phospho-histone H3 (Ser10)	9701	Rabbit	1∶100	Cell Signaling	P-IHC
phospho-Smad1/5/8	9511	Rabbit	1∶50	Cell Signaling	P-IHC
phospho-Smad2	3101	Rabbit	1∶50	Cell Signaling	P-IHC
phospho-Src Family (Tyr416)	2101	Rabbit	1∶25	Cell Signaling	P-IHC
Tgn138	2F7.1	Mouse IgG1	1∶50	Affinity Bioreagents	IF
Zdhhc21	299	Rabbit	1∶100–500	Custom Eurogentec	P-IHC, IF, WB

**Table 2:** Details of secondary antibodies used in this study.

Antigen	Host Species	Dilution	Source	Notes
ECL α-Mouse IgG, HRP-conjugated	Sheep	1∶10000	GE Healthcare UK Ltd	WB
ECL α-Rabbit IgG, HRP-conjugated	Sheep	1∶10000	GE Healthcare UK Ltd	WB
TRITC-conjugated α-Rabbit IgG	Donkey	1∶250	Jackson Immunoresearch	P-IHC, IF
FITC-conjugated α-Rabbit IgG	Donkey	1∶250	Jackson Immunoresearch	P-IHC, IF
Alexa 488-conjugated-α-Mouse	Donkey	1∶500	Molecular Probes	P-IHC, IF
Alexa 594-conjugated-α-Mouse	Donkey	1∶500	Molecular Probes	P-IHC, IF
Alexa 594-conjugated-α-Rabbit	Donkey	1∶500	Molecular Probes	P-IHC, IF
Alexa 488-conjugated-α-Rat	Donkey	1∶500	Molecular Probes	P-IHC, IF
Biotin-conjugated-α-Rabbit	Donkey	1∶400	Jackson Immunoresearch	P-IHC, IF

### BrdU labeling

For BrdU labeling experiments, 2 age- and gender-matched mice of each genotype were injected with 50 µg BrdU/g body weight and sacrificed after 2 hrs. Skin sections were dewaxed, subjected to proteinase K antigen retrieval, followed by HCl denaturation and neutralization, before incubation with anti-BrdU antibody (BD). For indirect colorimetric visualization, a biotinylated donkey anti-mouse secondary antibody (Jackson Labs) and Vectastain Universal Elite ABC Kit (Vector Laboratories) were used, followed by NovaRed substrate (Vector Laboratories) according to manufacturer's protocol. A proliferative index was calculated by counting the number of positive cells divided by the total number of nuclei within the epidermal compartment, in each of ten fields at 10× magnification, and the average index per field was calculated. Statistical significance was calculated using a two-tailed Student's t-test.

## Supporting Information

Figure S1 Localization and function of Zdhhc21 is altered by mutations of cysteines within DHHC consensus core. (A) Schematic of mutations in Zdhhc21. In addition to the *dep* deletion in C-terminal intracellular tail, several point mutations were generated by disrupting key cysteine residues within the DHHC domain. Another mutation, L91F, close to the DHHC domain was identified from an archive of ENU-mutagenised sperm from Harwell. However unlike mutations in the critical cysteines, this mutant protein was correctly localized and exhibited normal PAT activity. Mice homozygous for this mutation had normal hair. (B–G) Localization of HA-tagged Zdhhc21 cDNAs transfected into NIH-3T3 cells (anti-HA red) compared to cis-Golgi marker GM130 (green). Wild type and L91F strongly co-localize with GM130, whereas mutations within DHHC domain disrupt localization similar to dep. (H) Zdhhc21 protein variants which disrupt localization abrogate autopalmitoylation responses using ABE chemistry and pulled down by streptavidin agarose beads and resolved by SDS-PAGE [47]. Portions not pulled down were also resolved by SDS-PAGE as loading control (I).
(1.66 MB TIF)

Figure S2 Characterization of Zdhhc21 expression in skin. Expression of *Zdhhc21* mRNA (B,D,E,G,J) and protein (A,C,F,H,I,K,). (A) E16.5 vibrissae follicle (Zdhhc21: green, p63: red). (B,C) P24 dorsal control skin. (D–F) P35 dorsal follicles of *dep* (D) and wild type (E), show similar levels and patterns of transcript, as observed with Zdhhc21 antibody (F). (G–I) While *Zdhhc21* mRNA and protein expression is similar in the lower portions of P63 dorsal follicles (G,H), only protein can be detected in the upper (I) portions of the isthmus (I) but not in the bulge, sebaceous glands or IFE. (J–L) In telogen, (P21) wild-type dorsal skin shows no expression of *Zdhhc21* mRNA (J) while some antibody staining is detected in the isthmus (K), which is specifically blocked by pre-incubating the antibody with the blocking peptide (L).
(4.99 MB TIF)

Figure S3 Cyclic expression of Zdhhc21 during postnatal hair cycle in wild-type and dep mutant follicles. Expression of Zdhhc21 (red) and Gata3 (green) during catagen (P14 A,B), telogen (P21 C,D), initiation of anagen (P24 E,F), early anagen (P28 G,H) and late anagen (P35 I,J) in wild-type (A,C,E,G,I) and *dep* follicles (B,D,F,H,J). Expression of Zdhhc21 is limited to the post-mitotic lineages of IRS and cuticle of both control and dep anagen and catagen follicles.
(6.63 MB TIF)

Figure S4 Aberrant epidermal proliferation during anagen contributes to *dep* hyperplastic interfollicular epidermis and sebaceous glands. Hematoxylin and eosin (A–D). Phosphohistone H3 (red, E–J) with Ki67 (green; I,J,). Significant differences in proliferation were not readily detectable at telogen (P21; A,B,E,F), or early (P28; C,D,G–J) anagen. However, quantitative BrDU labelling studies during anagen (P32) revealed a small but significant increase in proliferation in *dep* sebaceous glands and IFE (L), with a parallel decrease in proliferation in *dep* hair follicles (K). (**p<0.005, *p<0.05)
(4.18 MB TIF)

Figure S5 Aberrant epidermal differentiation in *dep* mutant skin. Wild-type (A–F) and *dep* (G–,L) P28 dorsal follicles. Expression of terminal differentiation markers (loricrin (red), p63 (green) (A,G); filaggrin (red) (B,H) is delayed in *dep* mutant skin. Ectopic Keratin 6 expression (K6 (red), Ki67 (green) (C,I) is not observed in *dep* interfollicular epidermis, but expression remains restricted to the infundibulum and inner root sheath of the hair follicle. Imbalance of proliferative and differentiation signals in *dep* basal IFE where increased nuclear phospho-ERK (phospho-P42/44 (red), Gata3 (green), (D,–D′,J–J′) is observed with reduced expression of Gata3, in contrast to wild type skin where high suprabasal phospho-ERK is associated with strong Gata3 expressing cells (D–D′, arrowheads). Aberrant elevated basal p42/44 signalling was confirmed with a second antibody (I–I′,K–K′). Despite expanded bulge region below the dilated infundibulum and overgrown sebaceous glands, the expression of K15 (green) remains restricted to the bulge (F,L). Nuclei were labelled with DAPI (blue:C,I) or TOTO-3 (blue:D–F,J–L).
(4.65 MB TIF)

Figure S6 Loss of Zdhhc21 function does not result in delays in selective barrier acquisition or keratinocyte terminal differentiation defects in embryonic *dep* epidermis. Wild-type (A–E) and *dep* mutant (F–J) late E16.5 embryos and E18.5 embryonic skins (C–E, H–J). (A,B,F,G) Dye exclusion assay showing similar range of barrier acquisition in a litter with wild-type and *dep* littermates from less advanced (A,F) to more established stages of barrier development (B,G). No difference in expression of terminal differentiation markers loricrin (C,H) and filaggrin (D,I) is detected between wild type and *dep* neonatal skin. Comparable Gata3 expression is observed in developing hair follicles and IFE of wild-type and *dep* neonatal skin (E–J).
(2.42 MB TIF)

Figure S7 Initiation of Wnt-dependent anagen responses is normal in *dep* mice but subsequent propagation is affected. Alkaline phosphatase staining (A,C,E,G) marks dermal papillae. Induction of first anagen at P24 (A–D) with strong dermal papilla Lef1 staining (red) (B,D) and few adjacent positive cells in epidermal hair germ is observed in both wild-type (A,B) and mutant (C,D) skin. Subsequent propagation of anagen responses is defective at P28 (E–L) where retarded *dep* follicles show little Lef1 signal in matrix (F,H) as well as reduced or absent Foxn1 (green,Zdhhc21 red; J,K) and AE13 (green, Zdhhc21 red; J,L) in hair shaft precursors. By P35 (M–P), although misshapen and misoriented, many *dep* follicles (O,P) are similar to control littermates (M,N) as shown by AE13 (green, Zdhhc21 red; M,O) and beta-catenin (green, K5 red; N,P).
(5.73 MB TIF)

Figure S8 Effective membrane targeting of Fyn during keratinocyte differentiation is compromised in *dep* mutant skin. Fyn localization in P32 anagen follicles (A–B′) and IFE (E–F′) with localization of active Src family kinases (including Src, Fyn, Yes and Lck) (C–D′,G–H′). Fyn expression is detected diffusely in the wild type bulb and becomes restricted to the membrane of differentiating IRS cuticle and some Henle's layers at the junction of the hair bulb and shaft. High levels of membrane associated active SFKs are seen throughout anagen hair follicle including dermal papilla, proliferative matrix, ORS and IRS lineages. In dep mutants, this membrane association of Fyn is greatly reduced/absent whilst active Src family kinase expression is largely unchanged. Fyn expression in the control IFE and IF becomes membrane restricted in suprabasal, differentiating keratinocytes, whilst membrane associated active Src family kinases can be seen throughout the basal and suprabasal IFE. Membrane associated Fyn and active SFKs is delayed in *dep* mutants. Arrowheads indicated areas of interest in merge and single channels.
(4.72 MB TIF)
