# Supplementary figures and images for: Palmitoylation Regulates Epidermal Homeostasis and Hair Follicle Differentiation
Source: PLoS Genet. 2009 Nov 26;5(11):e1000748. doi: 10.1371/journal.pgen.1000748 (PMC2776530; doi:10.1371/journal.pgen.1000748)

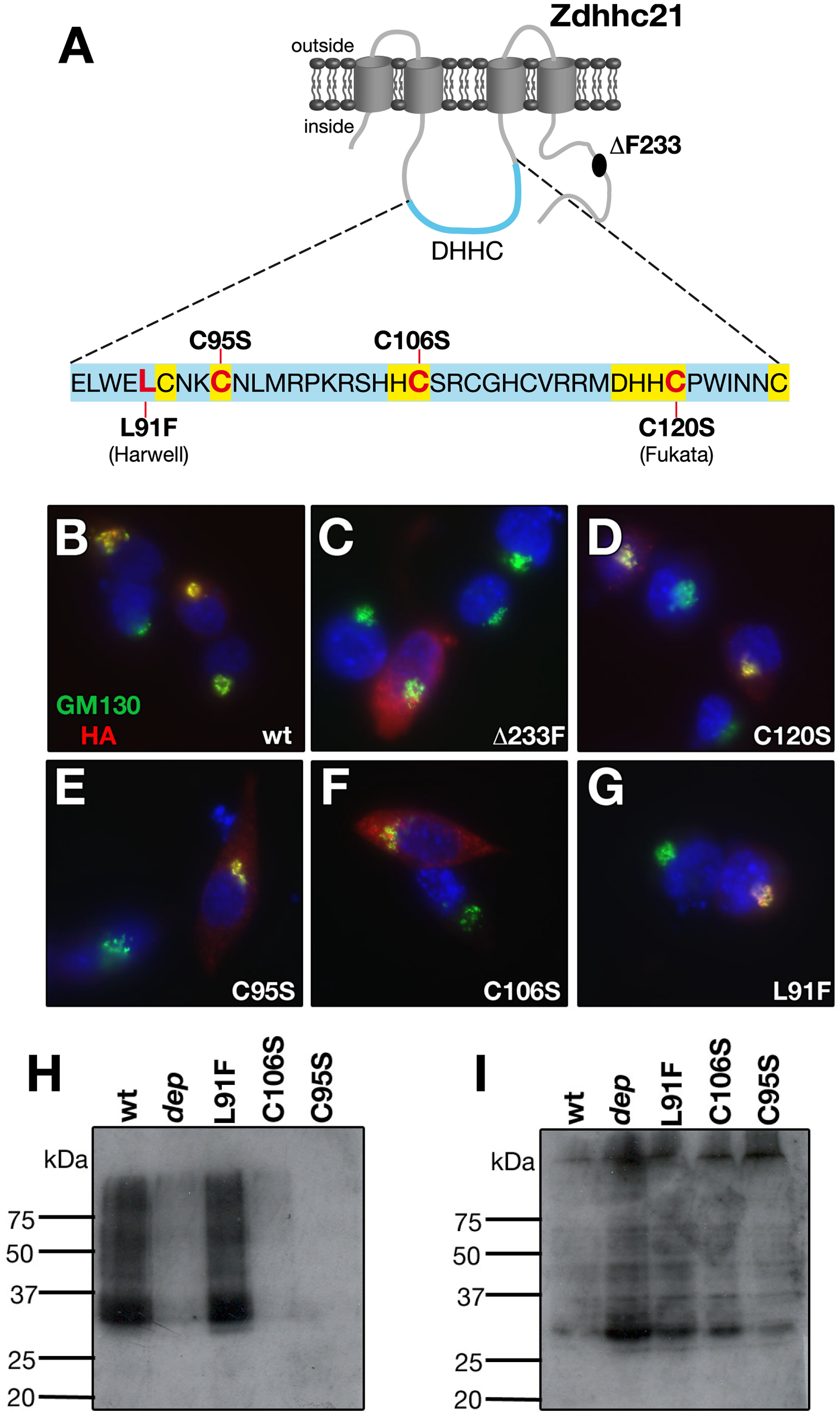

Supplement: Figure S1 — Localization and function of Zdhhc21 is altered by mutations of cysteines within DHHC consensus core. (A) Schematic of mutations in Zdhhc21. In addition to the dep deletion in C-terminal intracellular tail, several point mutations were generated by disrupting key cysteine residues within the DHHC domain. Another mutation, L91F, close to the DHHC domain was identified from an archive of ENU-mutagenised sperm from Harwell. However unlike mutations in the critical cysteines, this mutant protein was correctly localized and exhibited normal PAT activity. Mice homozygous for this mutation had normal hair. (B–G) Localization of HA-tagged Zdhhc21 cDNAs transfected into NIH-3T3 cells (anti-HA red) compared to cis-Golgi marker GM130 (green). Wild type and L91F strongly co-localize with GM130, whereas mutations within DHHC domain disrupt localization similar to dep. (H) Zdhhc21 protein variants which disrupt localization abrogate autopalmitoylation responses using ABE chemistry and pulled down by streptavidin agarose beads and resolved by SDS-PAGE [47]. Portions not pulled down were also resolved by SDS-PAGE as loading control (I). (1.66 MB TIF) [file pgen.1000748.s001.tif]

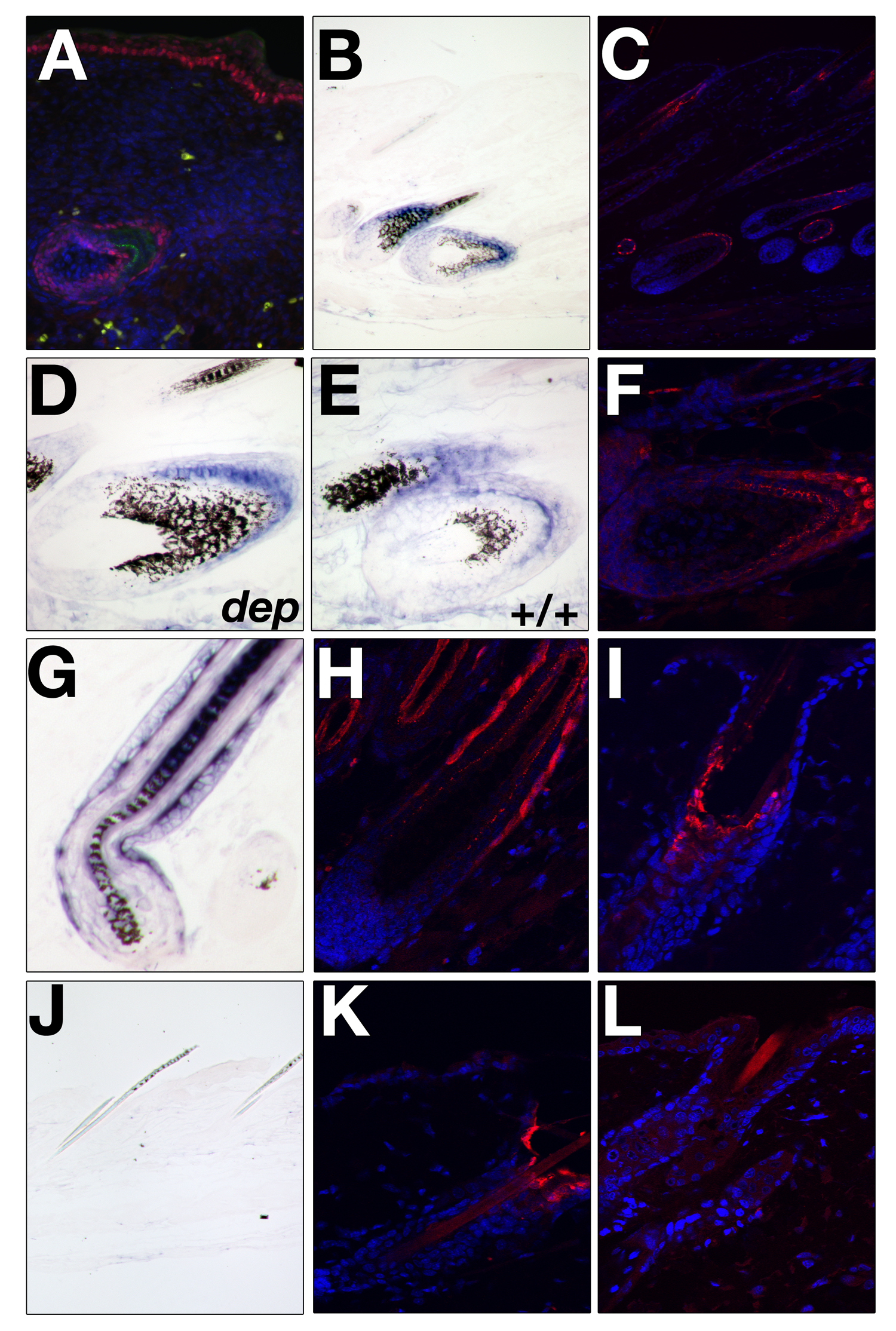

Supplement: Figure S2 — Characterization of Zdhhc21 expression in skin. Expression of Zdhhc21 mRNA (B,D,E,G,J) and protein (A,C,F,H,I,K,). (A) E16.5 vibrissae follicle (Zdhhc21: green, p63: red). (B,C) P24 dorsal control skin. (D–F) P35 dorsal follicles of dep (D) and wild type (E), show similar levels and patterns of transcript, as observed with Zdhhc21 antibody (F). (G–I) While Zdhhc21 mRNA and protein expression is similar in the lower portions of P63 dorsal follicles (G,H), only protein can be detected in the upper (I) portions of the isthmus (I) but not in the bulge, sebaceous glands or IFE. (J–L) In telogen, (P21) wild-type dorsal skin shows no expression of Zdhhc21 mRNA (J) while some antibody staining is detected in the isthmus (K), which is specifically blocked by pre-incubating the antibody with the blocking peptide (L). (4.99 MB TIF) [file pgen.1000748.s002.tif]

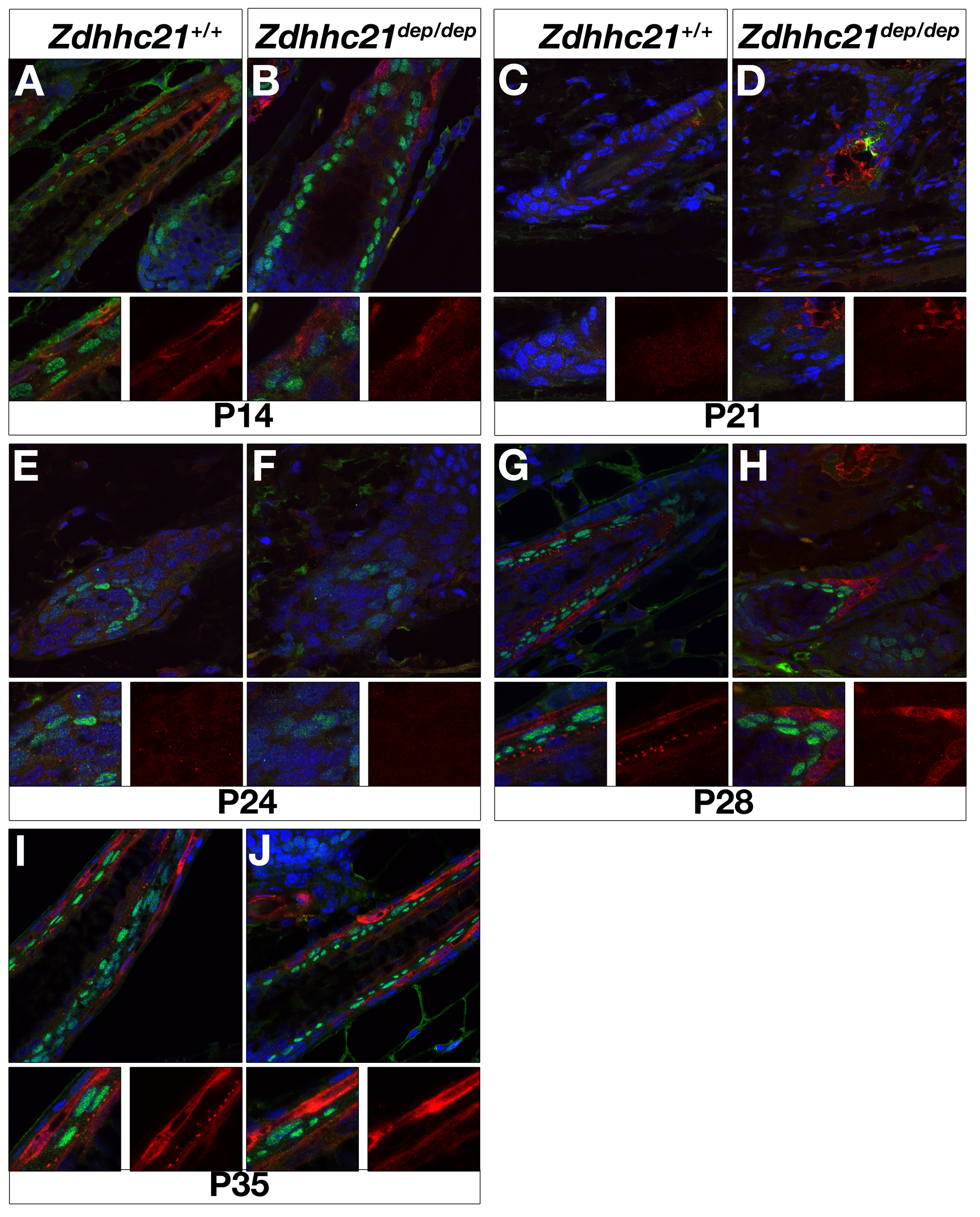

Supplement: Figure S3 — Cyclic expression of Zdhhc21 during postnatal hair cycle in wild-type and dep mutant follicles. Expression of Zdhhc21 (red) and Gata3 (green) during catagen (P14 A,B), telogen (P21 C,D), initiation of anagen (P24 E,F), early anagen (P28 G,H) and late anagen (P35 I,J) in wild-type (A,C,E,G,I) and dep follicles (B,D,F,H,J). Expression of Zdhhc21 is limited to the post-mitotic lineages of IRS and cuticle of both control and dep anagen and catagen follicles. (6.63 MB TIF) [file pgen.1000748.s003.tif]

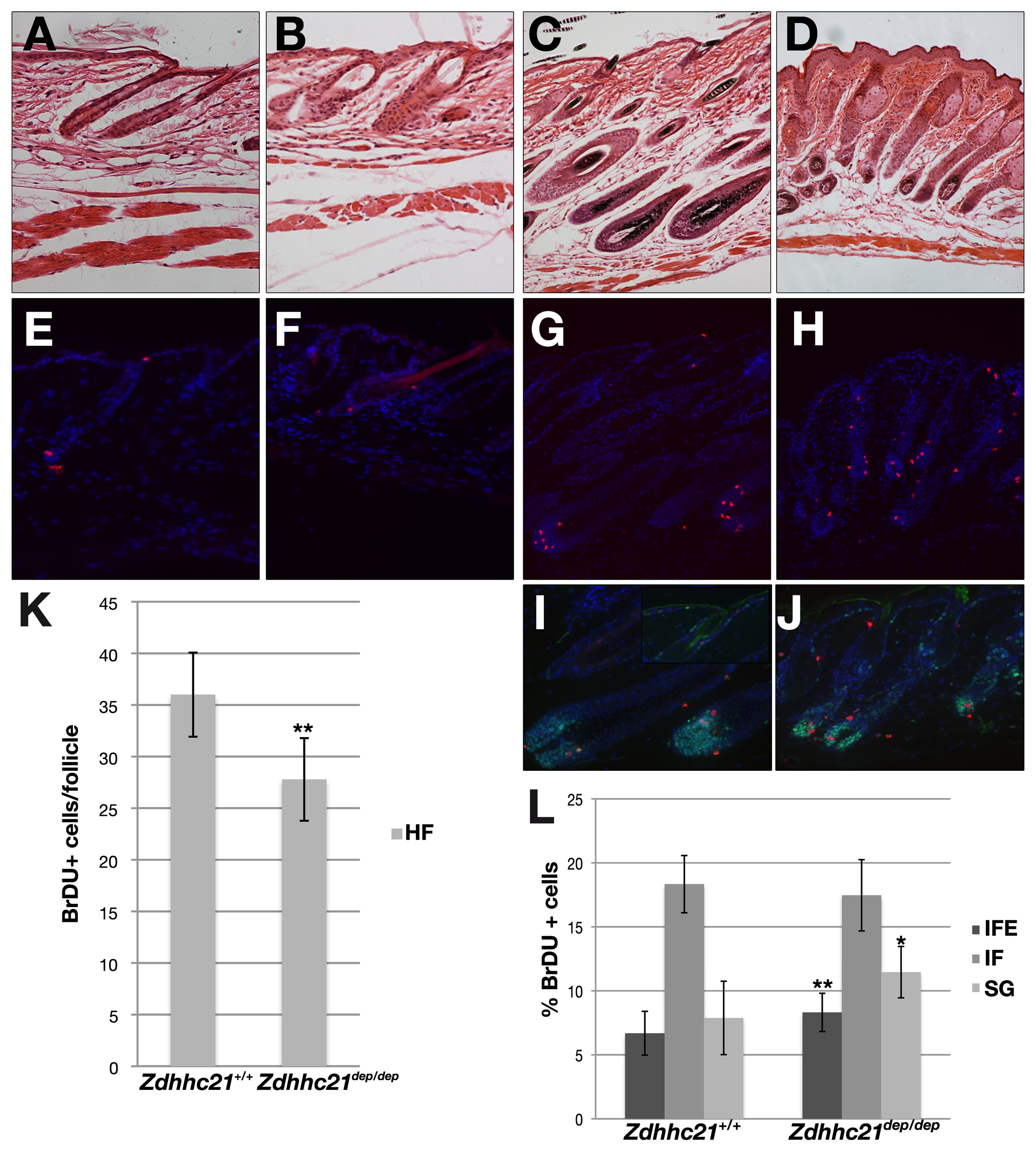

Supplement: Figure S4 — Aberrant epidermal proliferation during anagen contributes to dep hyperplastic interfollicular epidermis and sebaceous glands. Hematoxylin and eosin (A–D). Phosphohistone H3 (red, E–J) with Ki67 (green; I,J,). Significant differences in proliferation were not readily detectable at telogen (P21; A,B,E,F), or early (P28; C,D,G–J) anagen. However, quantitative BrDU labelling studies during anagen (P32) revealed a small but significant increase in proliferation in dep sebaceous glands and IFE (L), with a parallel decrease in proliferation in dep hair follicles (K). (**p<0.005, *p<0.05) (4.18 MB TIF) [file pgen.1000748.s004.tif]

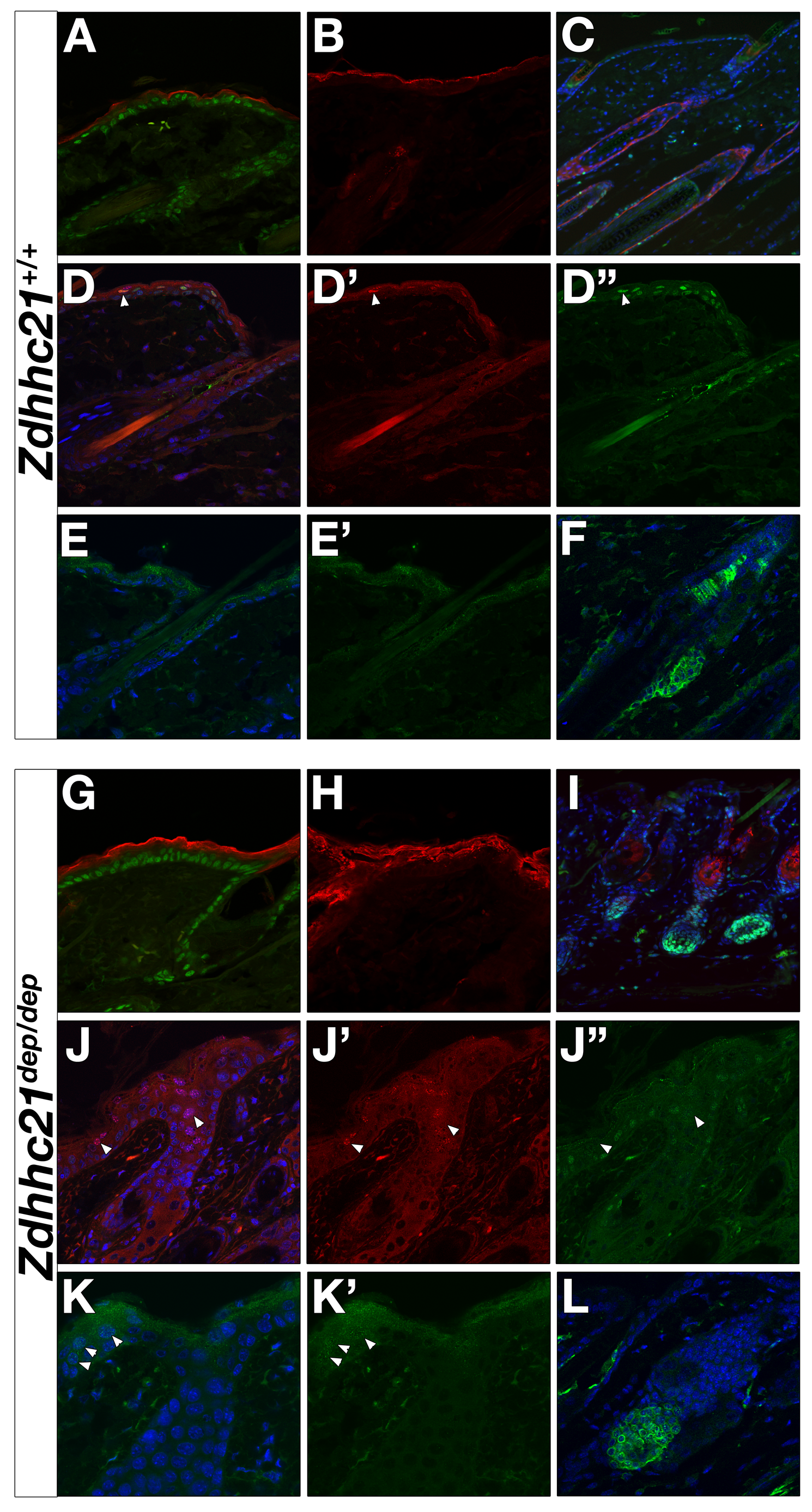

Supplement: Figure S5 — Aberrant epidermal differentiation in dep mutant skin. Wild-type (A–F) and dep (G–,L) P28 dorsal follicles. Expression of terminal differentiation markers (loricrin (red), p63 (green) (A,G); filaggrin (red) (B,H) is delayed in dep mutant skin. Ectopic Keratin 6 expression (K6 (red), Ki67 (green) (C,I) is not observed in dep interfollicular epidermis, but expression remains restricted to the infundibulum and inner root sheath of the hair follicle. Imbalance of proliferative and differentiation signals in dep basal IFE where increased nuclear phospho-ERK (phospho-P42/44 (red), Gata3 (green), (D,–D′,J–J′) is observed with reduced expression of Gata3, in contrast to wild type skin where high suprabasal phospho-ERK is associated with strong Gata3 expressing cells (D–D′, arrowheads). Aberrant elevated basal p42/44 signalling was confirmed with a second antibody (I–I′,K–K′). Despite expanded bulge region below the dilated infundibulum and overgrown sebaceous glands, the expression of K15 (green) remains restricted to the bulge (F,L). Nuclei were labelled with DAPI (blue:C,I) or TOTO-3 (blue:D–F,J–L). (4.65 MB TIF) [file pgen.1000748.s005.tif]

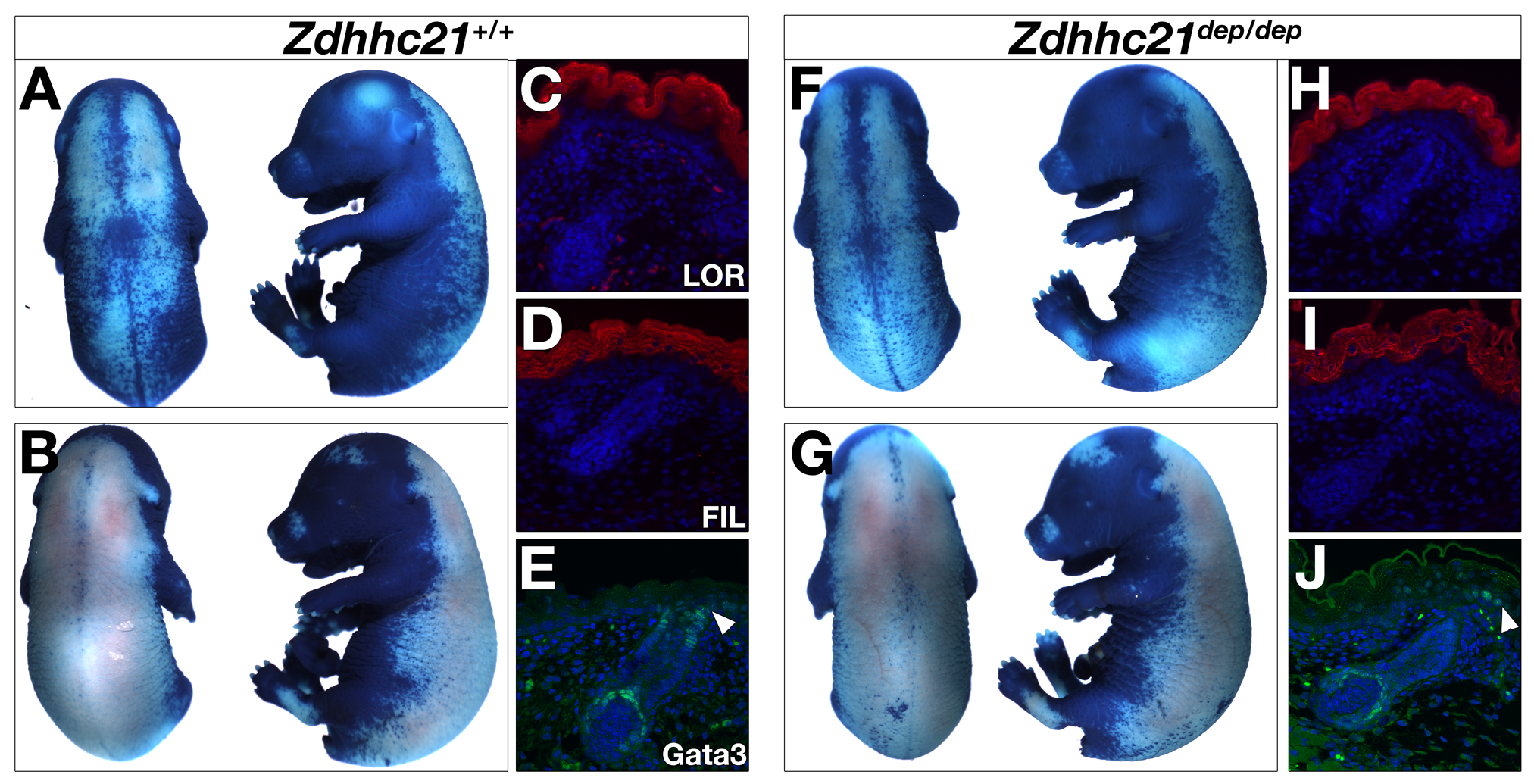

Supplement: Figure S6 — Loss of Zdhhc21 function does not result in delays in selective barrier acquisition or keratinocyte terminal differentiation defects in embryonic dep epidermis. Wild-type (A–E) and dep mutant (F–J) late E16.5 embryos and E18.5 embryonic skins (C–E, H–J). (A,B,F,G) Dye exclusion assay showing similar range of barrier acquisition in a litter with wild-type and dep littermates from less advanced (A,F) to more established stages of barrier development (B,G). No difference in expression of terminal differentiation markers loricrin (C,H) and filaggrin (D,I) is detected between wild type and dep neonatal skin. Comparable Gata3 expression is observed in developing hair follicles and IFE of wild-type and dep neonatal skin (E–J). (2.42 MB TIF) [file pgen.1000748.s006.tif]

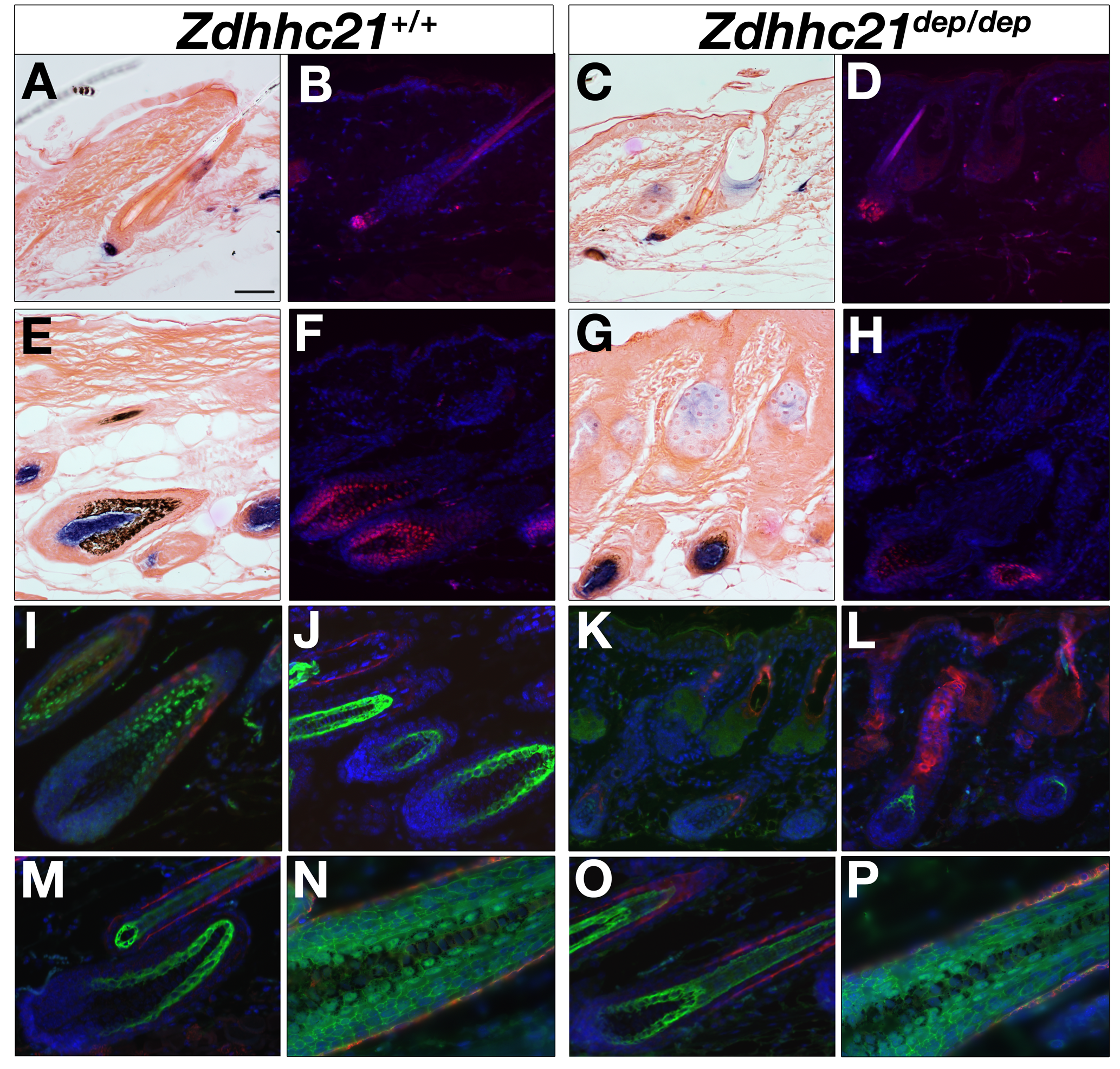

Supplement: Figure S7 — Initiation of Wnt-dependent anagen responses is normal in dep mice but subsequent propagation is affected. Alkaline phosphatase staining (A,C,E,G) marks dermal papillae. Induction of first anagen at P24 (A–D) with strong dermal papilla Lef1 staining (red) (B,D) and few adjacent positive cells in epidermal hair germ is observed in both wild-type (A,B) and mutant (C,D) skin. Subsequent propagation of anagen responses is defective at P28 (E–L) where retarded dep follicles show little Lef1 signal in matrix (F,H) as well as reduced or absent Foxn1 (green,Zdhhc21 red; J,K) and AE13 (green, Zdhhc21 red; J,L) in hair shaft precursors. By P35 (M–P), although misshapen and misoriented, many dep follicles (O,P) are similar to control littermates (M,N) as shown by AE13 (green, Zdhhc21 red; M,O) and beta-catenin (green, K5 red; N,P). (5.73 MB TIF) [file pgen.1000748.s007.tif]

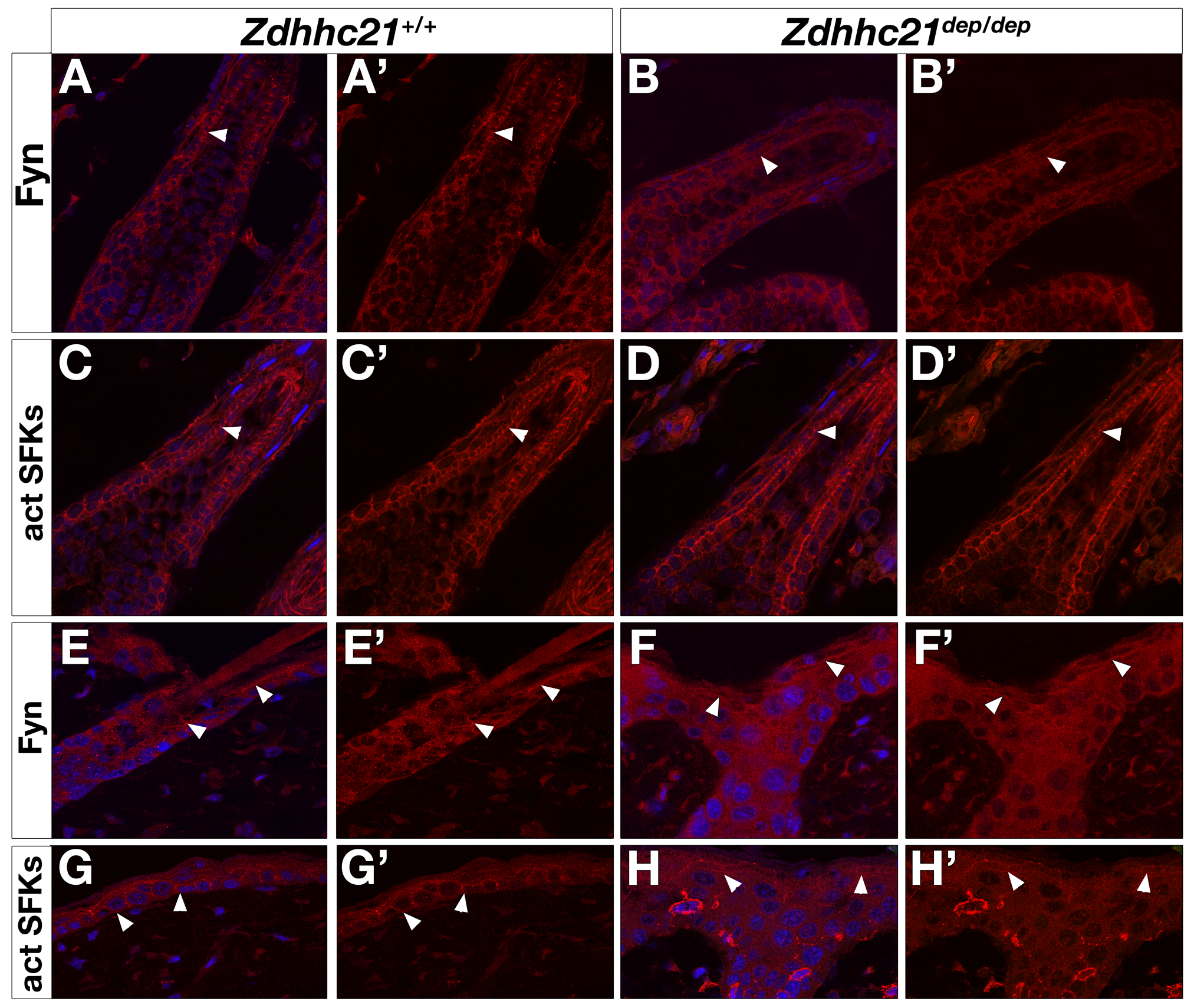

Supplement: Figure S8 — Effective membrane targeting of Fyn during keratinocyte differentiation is compromised in dep mutant skin. Fyn localization in P32 anagen follicles (A–B′) and IFE (E–F′) with localization of active Src family kinases (including Src, Fyn, Yes and Lck) (C–D′,G–H′). Fyn expression is detected diffusely in the wild type bulb and becomes restricted to the membrane of differentiating IRS cuticle and some Henle's layers at the junction of the hair bulb and shaft. High levels of membrane associated active SFKs are seen throughout anagen hair follicle including dermal papilla, proliferative matrix, ORS and IRS lineages. In dep mutants, this membrane association of Fyn is greatly reduced/absent whilst active Src family kinase expression is largely unchanged. Fyn expression in the control IFE and IF becomes membrane restricted in suprabasal, differentiating keratinocytes, whilst membrane associated active Src family kinases can be seen throughout the basal and suprabasal IFE. Membrane associated Fyn and active SFKs is delayed in dep mutants. Arrowheads indicated areas of interest in merge and single channels. (4.72 MB TIF) [file pgen.1000748.s008.tif]
